# Non-Water-Suppressed ^1^H MR Spectroscopy with Orientational Prior Knowledge Shows Potential for Separating Intra- and Extramyocellular Lipid Signals in Human Myocardium

**DOI:** 10.1038/s41598-017-16318-0

**Published:** 2017-12-04

**Authors:** Ariane Fillmer, Andreas Hock, Donnie Cameron, Anke Henning

**Affiliations:** 1 Institute for Biomedical Engineering, University and ETH Zurich, Gloriastr. 35, 8092 Zurich, Switzerland; 2 Physikalisch-Technische Bundesanstalt (PTB), Abbestr. 2–12, 10587 Berlin, Germany; 3 Department of Psychiatry, Psychotherapy and Psychosomatics, Hospital of Psychiatry, University of Zurich, Lenggstr. 31, 8032 Zurich, Switzerland; 4 Norwich Medical School, University of East Anglia, Norwich, NR4 7UQ UK; 5 National Institute on Aging, National Institutes of Health, MedStar Harbor Hospital, 3001 South Hanover Street, Baltimore, MD21225 Maryland USA; 6 Max Planck Institute for Biological Cybernetics, Max Planck Ring 11, 72076 Tuebingen, Germany

## Abstract

Conditions such as type II diabetes are linked with elevated lipid levels in the heart, and significantly increased risk of heart failure; however, metabolic processes underlying the development of cardiac disease in type II diabetes are not fully understood. Here we present a non-invasive method for *in vivo* investigation of cardiac lipid metabolism: namely, IVS-McPRESS. This technique uses metabolite-cycled, non-water suppressed ^1^H cardiac magnetic resonance spectroscopy with prospective and retrospective motion correction. High-quality IVS-McPRESS data acquired from healthy volunteers allowed us to investigate the frequency shift of extramyocellular lipid signals, which depends on the myocardial fibre orientation. Assuming consistent voxel positioning relative to myofibres, the myofibre angle with the magnetic field was derived from the voxel orientation. For separation and individual analysis of intra- and extramyocellular lipid signals, the angle myocardial fibres in the spectroscopy voxel take with the magnetic field should be within ±24.5°. Metabolite and lipid concentrations were analysed with respect to BMI. Significant correlations between BMI and unsaturated fatty acids in intramyocellular lipids, and methylene groups in extramyocellular lipids were found. The proposed IVS-McPRESS technique enables non-invasive investigation of cardiac lipid metabolism and may thus be a useful tool to study healthy and pathological conditions.

## Introduction

Cardiac magnetic resonance spectroscopy (CMRS) is becoming increasingly popular as a non-invasive probe of myocardial metabolism^1^, permitting healthy and pathological conditions to be studied using different endogenous nuclei. Of these, phosphorus (^31^P) is the most widely investigated, reflecting the crucial role of phosphorus metabolites in myocardial energetics. However, proton (^1^H) CMRS is starting to play a larger role in clinical research, as it permits visualisation of important molecular species including triglycerides and creatine (Cr). While the Cr resonance is an important clinical target, myocardial triglycerides are arguably of greater interest, as the healthy human heart obtains approximately 70% of its energy from oxidation of long-chain fatty acids^1^. Furthermore, elevated lipid concentrations in the heart are associated with type 2 diabetes mellitus^2,3^ and obesity^4^, as well as impaired diastolic function^2,5^ and a number of other disease conditions^6,7^. However, in order to gain deeper insights from lipid concentrations in cardiac muscle tissue concerning development of lipid metabolism related cardiac disease, it is necessary to distinguish intramyocellular lipids (IMCL) from extramyocellular lipids (EMCL). Note that some publications have referred to these as intracardiomyocellular and extracardiomyocellular lipids, ICCL and ECCL respectively^8^, but the terms IMCL and EMCL will be used throughout this work.

Applications of ^1^H MRS in human skeletal muscle have shown that up to four IMCL peaks and their corresponding EMCL peaks can be discerned^9–12^ (see supporting information Fig. S1.1), while high resolution nuclear magnetic resonance studies on triglycerides^13,14^ found as many as nine independent peaks or multiplets in the triglyceride spectrum^13,14^. The two most prominent lipid moieties are attributed to intra- and extracellular methylene protons, while the others originate from, variously: methyl protons; methylene protons in esters; and methylene protons adjacent to unsaturated and polyunsaturated bonds in fatty acids, as well as protons attached to glycerol^15,16^. Although several of these peaks have been successfully resolved and separated into their EMCL and IMCL portions in skeletal muscle, in the myocardium they are harder to distinguish, and are thus typically considered as a whole^4,8,17–22^. Accurate separation of the two sets of lipid moieties would be of great interest: first, to explore IMCL’s utility as a pathological marker and its role as an energy substrate^23^; and second, to investigate whether EMCL is metabolically significant, and not just an inert adipocyte depot^24^. To date, only the most prominent lipid resonances have been used for quantification, with some works considering only the highest resonance^25^, and others assuming constant area ratios between different lipid resonances^8^. Robust separation of lipid signals from the two different compartments in human subjects, as well as a comprehensive analysis of these myocardial lipids, has not been forthcoming. This can be attributed to relatively poor spectral quality in ^1^H CMRS, caused by a number of factors, including static field inhomogeneities, poor water suppression, inadequate localisation, and cardiac and respiratory motion^26^. Furthermore, the chemical shift of the EMCL peaks exhibits a dependence on muscle fibre orientation with respect to the main magnetic field^9,10^, sometimes leading to substantial overlap with the IMCL peaks, which have a fixed chemical shift. To achieve separate and accurate quantification of IMCL and EMCL, these challenges must first be surmounted.

Several methods have been applied to improve spectral quality in ^1^H CMRS, including: rapid single-breath-hold acquisitions^25^; prospective volume-tracking^27,28^; respiratory gating with pressure transducers, radar techniques, or navigator echoes^27,29–31^; and improved image-based B_0_ shimming to compensate for static field inhomogeneities across the heart^32,33^. All of these methods were applied with routine electrocardiogram (ECG) triggering. Just as important as acquisition strategies, post-processing methods can also improve the quality of ^1^H CMRS spectra, with frequency alignment and phase correction of individual signal averages serving to minimise line-broadening and phase-cancellation that result from cardiac motion and transient B_0_ inhomogeneities^34^. While frequency alignment and phase correction can improve spectral quality, they require a strong reference peak to be effective and, given ^1^H CMRS is commonly applied with water suppression, a strong peak may not be available. Moreover, water suppression could interfere with navigator signals, leading to poor respiratory gating. These issues can be avoided, and the robustness of ^1^H CMRS can be improved, through the use of a metabolite-cycled (MC) non-water-suppressed spectroscopy^34–36^ sequence: a two-shot subtraction technique where the water peak is retained as a reference for spectral correction.

This work introduces the inner-volume-saturated, metabolite-cycled point resolved spectroscopy (IVS-McPRESS) method for robust, high quality ^1^H CMRS in the human heart. This method is a combination of MC non-water-suppressed ^1^H CMRS with point resolved spectroscopy (PRESS)^37^ localisation and inner volume saturation (IVS)^38,39^ together with cardiac triggering, retrospective respiratory gating, sophisticated B_0_ shimming^33^, and post-processing methods^34^. Furthermore, conditions for the reliable resolution of the different lipid moiety signals are investigated, for use in both cardiac and skeletal muscle studies. Combination of this analysis with the IVS-McPRESS acquisition method will enable the separate investigation of IMCL and EMCL components, allowing for detailed examination of myocardial lipid metabolism.

## Methods

### Theory

Metabolite quantification of both ^1^H MR skeletal muscle spectra and ^1^H MR cardiac muscle spectra is complicated by effects induced by the fibre structure of the tissue. Regarding lipid quantification, so-called bulk susceptibility effects are of particular interest.

### Bulk Susceptibility Effects

**Figure 1:**
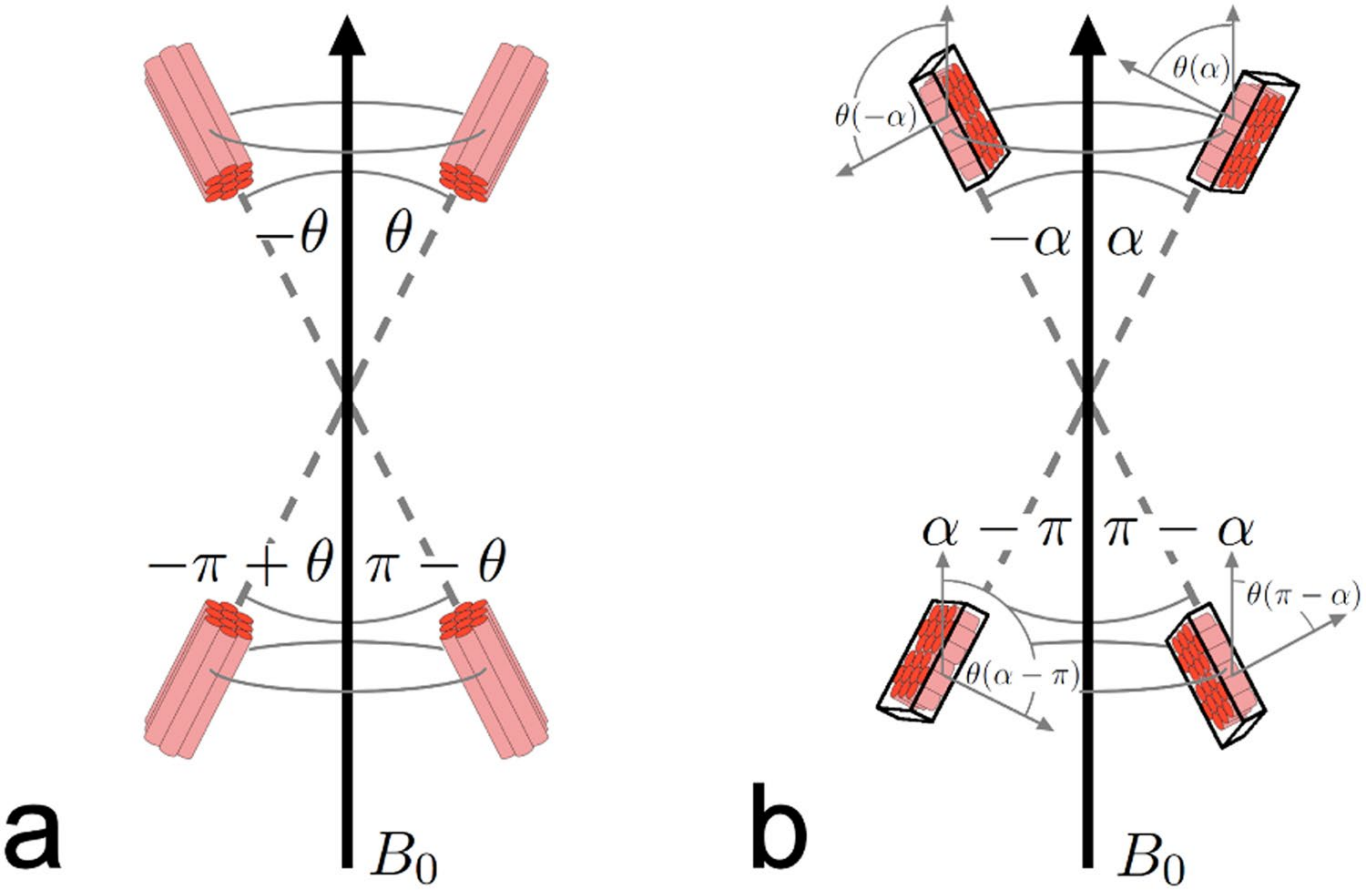
Voxel positioning with respect to fibre orientation and main magnetic field B_0_. The chemical shift of extramyocellular lipid (EMCL) resonances depends on the angle $\theta$ between the muscle fibres and the magnetic field (B_0_). (**a**) Since only the angle with the B_0_ axis is relevant, four angles $\theta$, $-\theta$, $\pi -\theta$, and $-\pi +\theta$ in the range $-\pi$ to $\pi$ can be considered equivalent and will result in the same ${\rm{\Delta }}{\omega }_{EMCL}$. (**b**) Assuming consistent voxel placement with respect to the muscle fibre orientation, the dependence can be examined with respect to the angle $\alpha$ between the voxel itself and B_0_. However, any offset between the two angles $\alpha$ and $\theta$ also needs to be accounted for, as does the possibility that the voxel and the muscular fibres are oriented differently with respect to the axis orthogonal to B_0_, which might result in $\theta$ not assuming the full range of values between $-\pi$ and $\pi$, while $\alpha$ is swept through the whole range from $-\pi$ to $\pi$. A more detailed explanation of this can be found in the supporting information. Note that this figure is intended as an illustration of how the different angles are related., and is not meant to imply that the myocardial fibres are oriented parallel to the shortest axis of the measurement voxel.

IMCL and EMCL have identical chemical compositions, but experience different chemical shifts (see supporting information Fig. S1.1); studies in skeletal muscle have shown this to be a result of bulk susceptibility effects^9,11^. While the resonance frequencies of IMCL signals are fixed, the chemical shift of EMCL depends on the angle, $\theta$, muscle fibres take relative to the main magnetic field (B_0_)^9,10^. Four of these angles are expected to give identical shifts: $\theta$, $-\theta$, $\pi -\theta$, and $-\pi +\theta$ (Fig. 1a). Working under the assumption that EMCL is confined between filamentary muscle cells to a tubular form, Boesch *et al*.^9^ calculated that the difference of the EMCL chemical shift with respect to the IMCL resonance frequency, ${\rm{\Delta }}{\omega }_{EMCL}$, ranges from −0.1 ppm to +0.21 ppm, with these extremes occurring when the tubular structures, and hence the muscle fibres, are aligned perpendicular or parallel to B_0_, respectively. Therefore, ${\rm{\Delta }}{\omega }_{EMCL}$ can be expressed in terms of $\theta$ as follows:

1 $${\rm{\Delta }}{\omega }_{EMCL}(\theta )=0.31\cdot {\cos }^{2}(\theta )-0.1,$$

an even, periodic function with four zero-crossings within the range $-\pi$ to $\pi$ and with maxima (0.21 ppm shift) and minima (−0.1 ppm shift) occurring at $\theta =n\cdot \pi /2$, where $n$ is a natural number. The total range of frequencies, ${\rm{\Delta }}{\omega }_{EMCL}(\theta )$ can assume, spans therefore 0.31 ppm.

Assuming consistent voxel positioning relative to cardiac fibre orientation, ${\rm{\Delta }}{\omega }_{EMCL}$ can also be expressed in terms of $\alpha$, the angle the voxel takes with B_0_ (Fig. 1b). Therefore, ${\rm{\Delta }}{\omega }_{EMCL}(\alpha )$ may be described by:

2 $${\rm{\Delta }}{\omega }_{EMCL}(\alpha )={\cos }^{2}(\rho )\cdot 0.31\cdot {\cos }^{2}(\alpha -{\alpha }_{\theta })-0.1,$$

where $\rho$ denotes the fibre orientation relative to the rotation plane, assuming the whole system of voxel and myocardial fibres could be rotated around an axis perpendicular to B_0_, and ${\alpha }_{\theta }$ is the fibre angle relative to the plane the voxel orientation spans with the rotation axis. While the heart is certainly not being rotated in the scanner, this theoretical concept describes all possible relative orientations between the voxel, the myocardial fibres, and B_0_. More details of this substitution are given in chapter 2 of the supporting information.

### MRS Pulse Sequence

**Figure 2:**
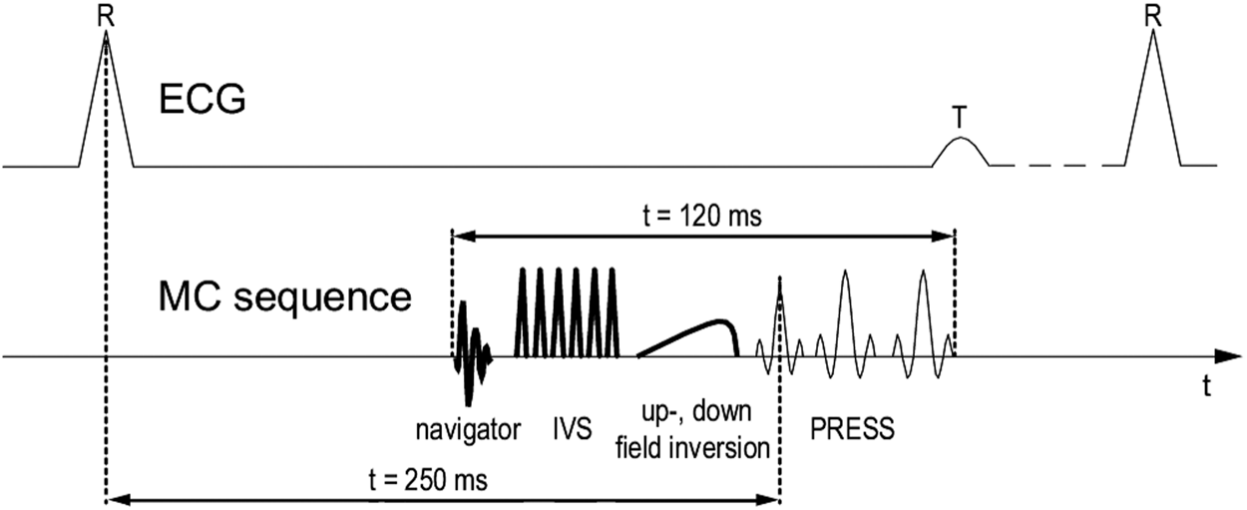
A sequence diagram of the proposed IVS-McPRESS technique. After the recording of a navigator signal to assess the breathing status of the volunteers, an inner volume suppression (IVS) scheme is applied to improve the robustness of the localisation. Then an upfield or downfield inversion pulse is applied in alternating measurements, inverting all upfield or all downfield metabolites, respectively, while leaving the water signal untouched. Finally, a point resolved spectroscopy (PRESS) localisation is employed. The sequence is initiated by an ECG trigger, with the excitation pulse occurring approximately 250 ms after that, with the total duration of all radio frequency pulses being 120 ms. By subtraction of an upfield and a downfield inverted measurement, a metabolite spectrum without water signal can be generated.

A PRESS^37^ localisation was employed in combination with the MC technique^35^. The MC method applies an upfield or downfield inversion pulse before alternate PRESS shots, inverting the metabolites upfield or downfield of the water peak, respectively, but leaving the water peak untouched. By subtracting the downfield-inverted spectra from the upfield-inverted spectra, a spectrum without water signal can be calculated, obviating the need for lengthy water-suppression techniques. By adding the downfield-inverted and upfield-inverted spectra, this technique allows the calculation of a pure water spectrum, from which water peak linewidths can be obtained without the need for an additional scan. In order to improve localisation and minimise the chemical shift displacement artefact, six inner-volume saturation (IVS)^38,39^ bands were also applied. This acquisition method will henceforth be referred to as IVS-McPRESS; a sequence diagram is displayed in Fig. 2.

### Data acquisition

**Figure 3:**
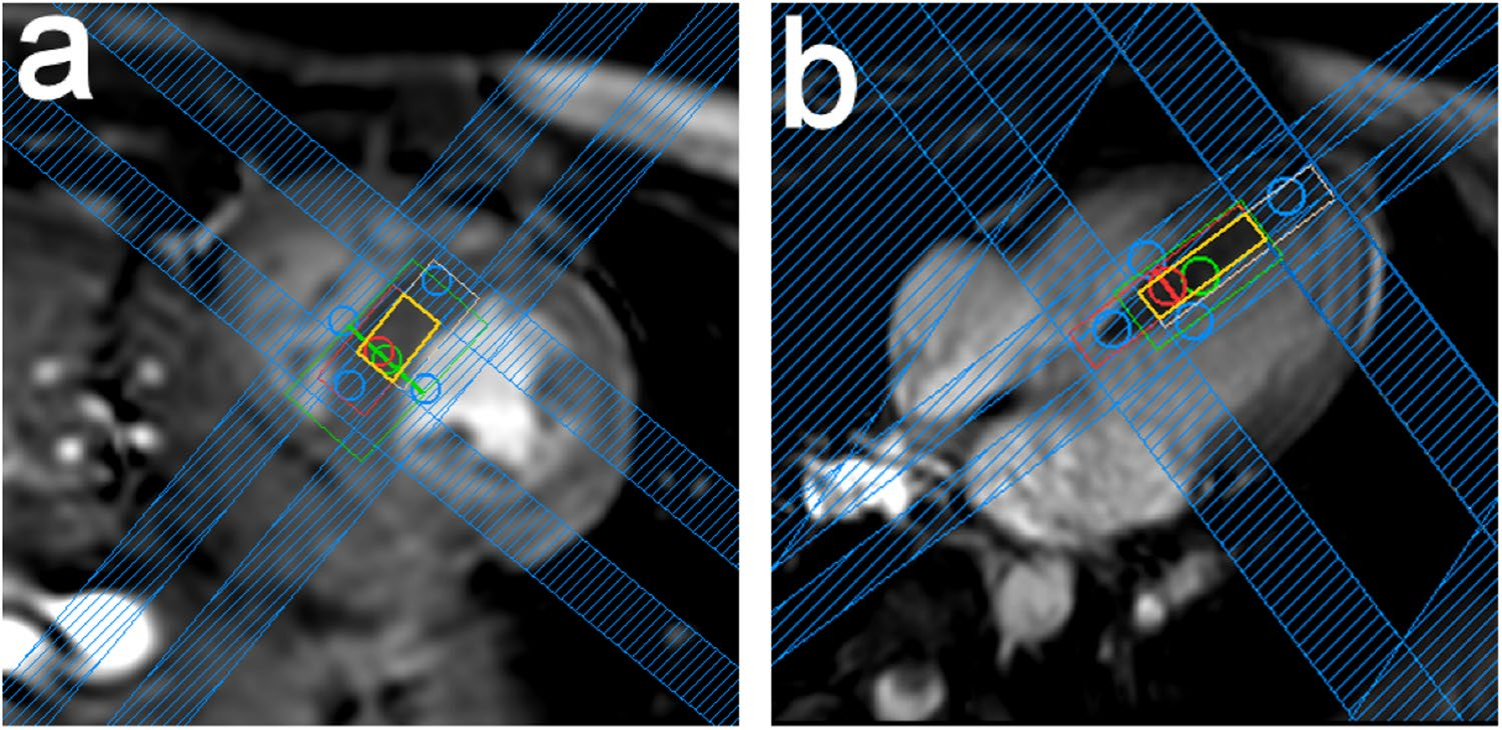
Voxel positioning. An end systolic short-axis view (**a**) and a quasi-4-chamber view (**b**) of a volunteer’s heart are shown. The spectroscopy voxel was placed in the interventricular septum, with the effective acquisition volume indicated in orange. The red and white boxes indicate the excitation volume at 4.6 ppm (water frequency) and at 2.0 ppm, respectively, which differ due to chemical shift displacement. Inner-volume saturation (IVS) bands (blue) are used to saturate areas of the voxel that are subject to only partial excitation in the desired frequency range, due to chemical shift displacement, thus increasing localisation robustness.

Measurements were performed using a 3 T Achieva MR system with a 32-channel cardiac receive array coil (Philips Healthcare, Best, NL) in 10 healthy female volunteers: median (range) age = 26 (21–30) years; median (range) body mass index (BMI) = 21.2 (17–27.3) kg/m^2^; median (range) heartrate = 58.5 (≈50 to ≈80) bpm. All volunteers gave written informed consent according to the guidelines of the cantonal ethics board of Zurich. All methods were performed in accordance with the relevant guidelines and regulations. Navigator-gated balanced steady-state free-precession cine images of short axis and quasi-four-chamber views of the heart were used to plan the spectroscopy voxel (Fig. 3) in the interventricular septum. ECG-triggered and respiratory-gated B_0_ maps were acquired to serve as a basis for B_0_ shim calculations using an image-based Shimtool^32,33^. Linear shim settings were optimised over the voxel of interest, and an automatically weighted region of less interest (ROLI) covering the whole heart was also considered during the calculation^33^. The IVS-McPRESS sequence was applied with an effective voxel size of 9.2 × 6.1 × 18.7 mm^3^, a minimum repetition time ≥3 heart beats, an echo time of 32 ms, and an acquisition time of 512 ms. Furthermore, it was ECG-triggered to end systole (trigger delay approx. 250 ms) and respiratory motion was measured using a navigator placed at the right hemi-diaphragm. Due to a lack of volume-based, ECG-triggered routines for flip angle optimisation, optimum flip angles were approximated manually by iterating through different transmit voltages for single shot non-water suppressed spectral acquisitions of the target voxel until the water peak intensity was maximised. The angle $\alpha$ between the voxel and B_0_ was read out from the parameter file (header file) of the acquired data.

A total of 256 averages were recorded within each IVS-McPRESS measurement, which took approximately 13 minutes, depending on the volunteer’s heartrate. The measurement was then repeated until the end of the allocated 2-hour timeslot. This led to a minimum of 512 averages acquired per subject, except for one case, where only 448 averages were acquired; however, the latter data set showed an excellent signal-to-noise ratio (SNR) of 25, as compared to the mean (standard deviation, SD) SNR of 21.4 (10.6), and was thus retained for analysis.

### Post-processing

MRecon (Gyrotools, Zurich, CH) was used to access raw data and to perform singular value decomposition coil combination. The individual signal acquisitions were zero-filled to twice their original size prior to phase- and eddy-current-correction, and frequency-aligned (see supporting information Fig. S3.1) to the unsuppressed water peak. Afterwards, each acquisition was truncated to 100 ms, at which point the signal has completely decayed, to avoid noise amplification due to overlong acquisition times. Each truncated acquisition was then zero-filled a second time to double the lengths of the original signal acquisition. Afterwards, the upfield-inverted spectra and the downfield-inverted spectra were averaged individually and subtracted from one another to produce a full IVS-McPRESS spectrum. A second spectrum was then calculated where all FIDs acquired with a navigator offset of more than 8 mm from the expiration position were discarded prior to averaging. If the latter data showed lower SNR than the spectrum calculated from all acquired averages, it was assumed that the volunteer primarily performed abdominal breathing and heart displacement due to respiratory motion was negligible. Hence, of the two calculated spectra, only the one with the largest SNR was retained for further analysis, where SNR is defined as the maximum signal within the analysis window of the spectrum, minus the baseline, divided by twice the root mean square of the residual, as reported by LCModel^40^.

### Quantification

In order to fit and quantify the spectral components of the acquired spectra the standard tool LCModel^40^ was used with the ‘muscle-5’ setting, assuming that - as a first approximation - cardiac muscle tissue has a similar composition to skeletal muscle tissue. LCModel, with the ‘muscle-5’ setting, is widely-used for quantification of ^1^H skeletal muscle spectra; it does not require the input of a basis set, and is thus less prone to user-dependent results than other tools.

IMCL resonances (IMCL09, CH_3_ at 0.9 ppm; IMCL13, CH_2_ at 1.3 ppm; and IMCL21, CH_2_ close to double bindings in lipid chain at 2.1 ppm) and EMCL resonances (EMCL11, CH_3_ at 1.1 ppm; and EMCL15, CH_2_ at 1.5 ppm) were analysed, along with the sum of CH_2_ next to the glycerin from IMCL and CH_2_ close to double bindings in lipid chain from EMCL at 2.3 ppm (I/E23), trimethyl-ammonium (TMA) compounds at 3.2 ppm, and taurine (tau) at 3.4 ppm. Relative concentrations were calculated for all metabolites using the creatine resonance (Cr30) at 3.0 ppm as a reference. Since the Cramér–Rao lower bounds (CRLBs) of the creatine signal at 3.9 ppm (Cr39), as calculated by LCModel, were very high compared to the CRLBs of other metabolites (see Table 1), the Cr39 resonance was excluded from further quantification analysis.

**Table 1:** Metabolite/Cr ratios and Cramér-Rao-Lower-Bounds (CRLB) from LCModel given for each volunteer.

volunteer	demographics		IMCL13		EMCL15		IMCL09		EMCL11		IMCL21		I/E23		TMA		tau		Cr39	
	age [y]	BMI [kg/m^2^]	Cr ratio	CRLB [%]	Cr ratio	CRLB [%]	Cr ratio	CRLB [%]	Cr ratio	CRLB [%]	Cr ratio	CRLB [%]	Cr ratio	CRLB [%]	Cr ratio	CRLB [%]	Cr ratio	CRLB [%]	Cr ratio	CRLB [%]
1	23	20.18	1.45	5	1.39	5	—	—	0.25	20	0.33	12	1.33	7	0.49	7	0.47	10	0.34	12
2	21	27.34	2.47	3	1.24	5	0.22	16	0.25	15	1.42	7	1.50	7	0.65	6	0.38	17	0.05	52
*3*	*25*	*19.14*	*3.82*	*1*	*0.06*	*36*	*0.80*	*7*	*0.06*	*28*	*0.62*	*17*	*2.19*	*8*	*0.95*	*3*	*0.53*	*10*	—	—
4	26	22.58	3.30	2	0.49	15	0.63	4	0.07	30	0.83	9	1.94	6	0.75	3	0.47	8	0.04	20
5	27	23.80	4.17	3	0.72	13	0.66	8	0.52	16	1.15	15	2.51	9	0.92	3	0.41	12	0.02	76
6	28	17.01	2.13	1	0.17	17	0.33	6	0.13	13	0.42	8	2.56	3	0.83	3	0.22	12	0.11	15
7	27	22.21	2.94	3	0.19	37	0.70	8	0.25	22	1.11	9	2.89	5	0.52	7	0.40	12	0.50	12
8	25	17.26	1.18	2	0.06	26	0.33	5	0.01	83	0.44	7	1.90	3	0.76	3	0.27	9	0.12	11
9	26	19.03	1.93	2	0.26	8	0.38	6	0.14	13	0.96	4	1.43	4	0.69	3	0.24	12	0.02	43
*10*	*30*	*22.48*	*2.35*	*2*	*0.22*	*17*	*0.83*	*5*	*0.02*	*75*	*1.47*	*5*	*2.11*	*4*	*0.69*	*3*	*0.18*	*15*	*0.00*	*604*
**Mean (SD)**	26.33 (2.00)	21.10 (3.20)	2.57 (0.98)	2.40 (1.17)	0.48 (0.48)	24.10 (22.54)	0.54 (0.23)	7.22 (3.56)	0.17 (0.15)	31.50 (25.77)	0.88 (0.41)	9.30 (4.19)	2.04 (0.52)	5.60 (2.12)	0.72 (0.15)	4.10 (1.79)	0.36 (0.12)	11.70 (2.71)	0.13 (0.17)	93.89 (192.63)

The *italic* cells indicate the volunteers with the highest and lowest angle $\alpha$, respectively, which were excluded from analysis with regard to lipid quantification. Means and standard deviations (SD) over all volunteers are given in the bottom row.

Finally, the metabolite/Cr ratios were analysed with respect to the BMI of the volunteers. Pearson’s correlation coefficient was determined in MATLAB (The Mathworks, Natick, MA, USA) for all identified metabolites in order to identify any statistically significant correlations.

### IMCL and EMCL Signal Separation

The frequency shift, ${\rm{\Delta }}{\omega }_{EMCL}$, of the largest EMCL signal, EMCL15, with respect to the corresponding IMCL signal, IMCL13, was extracted from the LCModel fit results. To investigate the dependence of ${\rm{\Delta }}{\omega }_{EMCL}$ on $\alpha$, the angle between the voxel and the main magnetic field, the parameters of equation (2) were fitted to the data. To verify this model is appropriate to describe the observed angulation dependence of the chemical shift of EMCL, adjusted ${R}^{2}$values and corrected Akaike information criterion (AICc) values were obtained for this model and a linear regression model that served as null hypothesis.

### Prior Knowledge for Quantification

To investigate the utility of the angular dependence of EMCL chemical shifts as prior knowledge for quantification, the spectra were fitted again using an undocumented parameter in LCModel, ‘chsimu’, which allowed the chemical shifts of the four major lipid components, IMCL13, EMCL15, IMCL09, and EMCL11, to be fixed, independent of their usual bounding conditions. During this procedure, the chemical shift of the most prominent resonance, ${\omega }_{IMCL13}$, was fixed to the value determined during the first fitting session, as this was deemed to be the most reliable frequency value. The chemical shift of the IMCL09, ${\omega }_{IMCL09}$, resonance was then calculated by:

3 $${\omega }_{IMCL09}={\omega }_{IMCL13}-0.4\,\text{ppm},$$

and the chemical shift of the EMCL15 and EMCL11 resonances, ${\omega }_{EMCL15}$ and ${\omega }_{EMCL11}$, were calculated by:

4 $${\omega }_{EMCL15}={\omega }_{IMLCL13}+{\rm{\Delta }}{\omega }_{EMCL},$$

5 $${\omega }_{EMCL11}={\omega }_{IMCL09}+{\rm{\Delta }}{\omega }_{EMCL},$$

using ${\rm{\Delta }}{\omega }_{EMCL}$ as determined from equation (2) with the obtained fit parameters for $\rho$ and ${\alpha }_{\theta }$, and values for $\alpha$ read from the parameter file.

The newly obtained metabolite/Cr ratios were again investigated with respect to BMI, and Pearson’s correlation coefficients were determined to identify statistically significant correlations.

## Results

**Figure 4:**
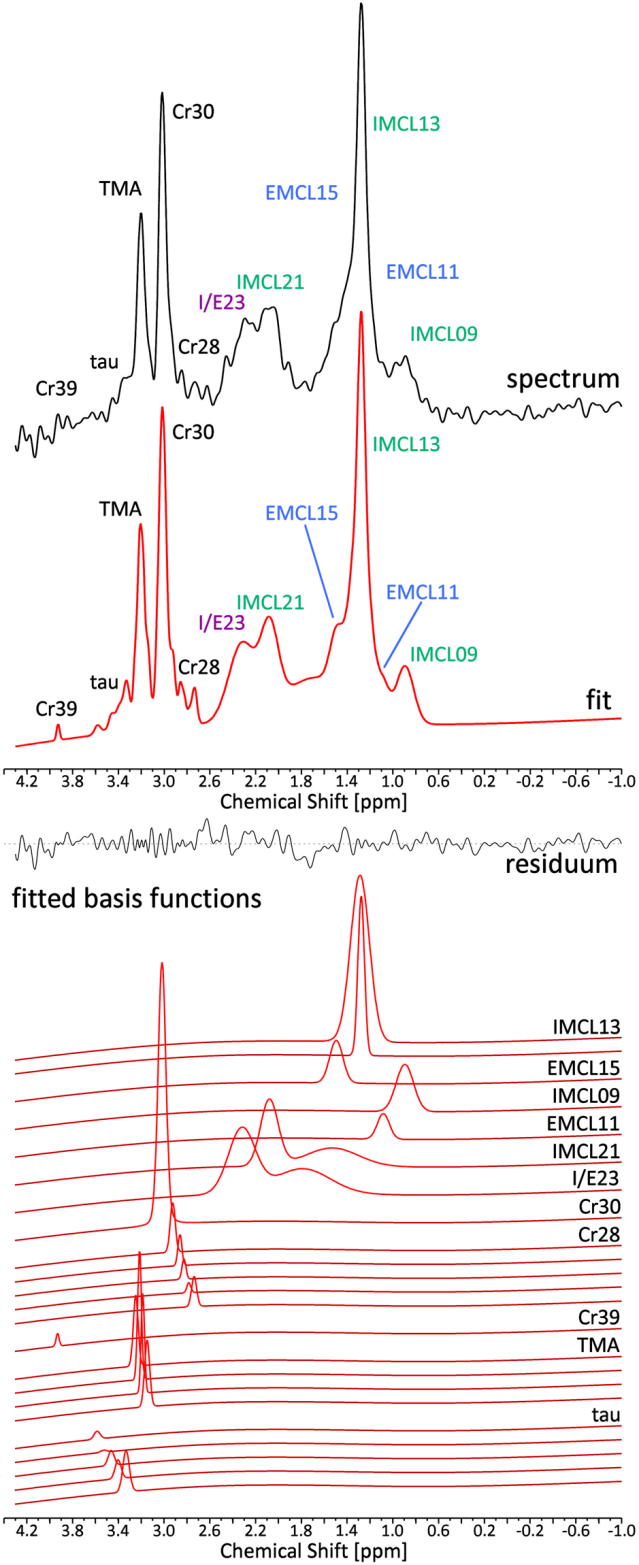
Measured ^1^H MR spectrum from the myocardium of a volunteer. The measured spectrum is shown (black, top panel) along with the fit (red, top panel). Different lipid components are indicated in green (intramyocellular lipids, IMCL) and blue (extramyocellular lipids, EMCL). The resonance from the IMCL resonance at 2.3 ppm and from the EMCL resonance corresponding to IMCL21, but shifted due to susceptibility effects to 2.3 ppm are approximated with only one basis set by LCModel. The mixed signal I/E23 of these two moieties is marked in purple. The middle panel displays the residuum of the LCModel fit. The different fitted basis functions from LCModel are shown in the bottom panel with the assigned metabolites on the right. Several metabolites are fitted as the sum of different slightly shifted peaks by LCModel to take effects on line shape, such as dipolar coupling, into account. In this case 1024 averages were acquired. Since respiratory motion effects did not have a negative effect on the spectrum’s SNR and line shape, no averages were discarded.

The mean (SD) of the water peak linewidths over all volunteers was 18 (1) Hz before correction and was further reduced to 12 (1) Hz with frequency alignment (see supporting information Fig. 3.1), and phase- and eddy-current-correction. A representative spectrum is displayed in Fig. 4, together with the LCModel fit and the fitted basis functions. The mean (SD) SNR of all measurements was 21 (11). Table 1 displays metabolite/Cr ratios and CRLBs as calculated by LCModel.

### IMCL and EMCL Signal Separation

**Figure 5:**
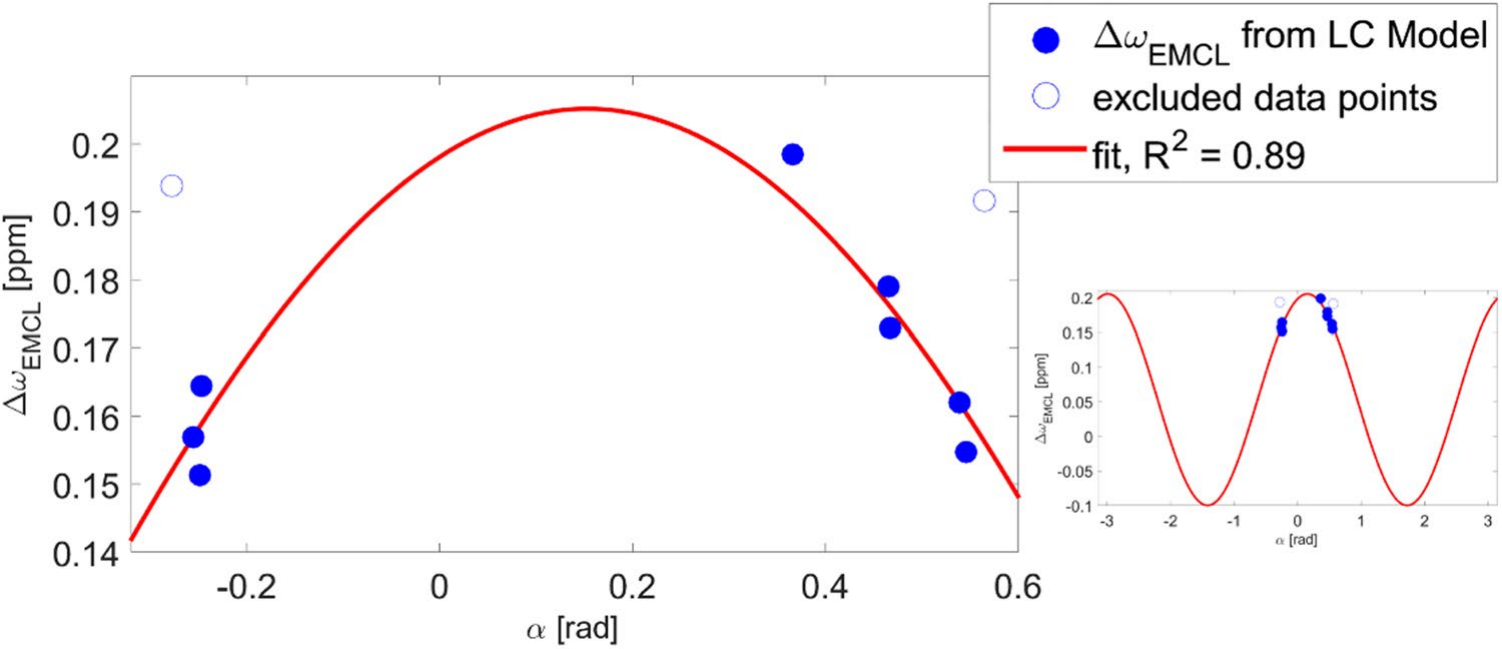
The angular dependence of lipid peak separation. Chemical shift difference of EMCL15 with respect to IMCL13, ${\rm{\Delta }}{\omega }_{EMCL}$ plotted over $\alpha$, the angle between the voxel and B_0_. A fit of equation (2) is indicated by the red curve. Calculated fit parameters are ${\alpha }_{\theta }=0.15\,{\rm{rad}}$ and $\rho =0.125\,{\rm{rad}}$, and the corresponding coefficient of determination, ${R}^{2}$, is 0.89. Two outliers (open circles), acquired with the largest and smallest angle $\alpha$, respectively, were excluded from the fit and from all following analysis, since the chemical shift range of the LCModel fit is constrained and is likely unable to correctly fit the frequency position of the peaks in question. The smaller plot on the right displays the course of the fit of equation (2) in the range from −$\pi$ to $\pi$.

In Fig. 5, ${\rm{\Delta }}{\omega }_{EMCL}$ is plotted versus $\alpha$, together with a fit of equation (2), which shows the dependence of the theoretical frequency shift of EMCL on $\alpha$. Clearly, the ${\rm{\Delta }}{\omega }_{EMCL}$ of the spectra closely follows the angular dependence given by equation (2), with a coefficient of determination, ${R}^{2}$, of 0.89. The calculated fit parameters were ${\alpha }_{\theta }=0.15{\rm{rad}}$ (${\alpha }_{\theta }=8.6^\circ$) and $\rho =0.13{\rm{rad}}$ ($\rho =7.2^\circ$). The two data points associated with the largest and the smallest $\alpha$, however, are clear outliers, represented by open circles. Given that the frequency shift of EMCL15 is expected to depend on $\alpha$, as per equation (2), and the other data points in Fig. 5 follow this relationship, the frequency shifts of the outlier data are expected to be significantly smaller than the values shown. In fact, in these outlier data we expect the EMCL15 resonances to show the smallest ${\rm{\Delta }}{\omega }_{EMCL}$ among all acquired spectra. By visual inspection of the spectra in question, a small ‘shoulder’ was apparent on the IMCL13 peak at the predicted frequency for EMCL15 (see supporting information Fig. 4.1). The reason this shoulder may not have been fitted is that the fit parameter that determines the frequency shift of the basis functions within LCModel is internally constrained, with the constraint values depending on several properties of the acquired spectrum, like SNR and linewidth. Furthermore, triglyceride spectra exhibit an additional low intensity spectral component at 1.6 ppm^13,14,16^, stemming from the methylene protons next-nearest to the glycerol in IMCL, which is not included in the fitting model used by LCModel. Indeed, the two outlier spectra showed fitted EMCL15 peak areas of the same order of magnitude as the noise, and it is therefore likely that the EMCL15 basis function was fitted either to noise or to the very low intensity signal of IMCL at 1.6 ppm in these cases. Hence, these two spectra were excluded from further analysis.

The remaining eight spectra, in which ${\rm{\Delta }}{\omega }_{EMCL}$ was close to theoretically derived values, were acquired with a voxel angle $\alpha$ within the range of $-0.26{\rm{rad}} < \alpha  < 0.56{\rm{rad}}$ ($-15^\circ  < \alpha  < 32^\circ$), which translates to roughly $-24.5^\circ  < \theta  < 24.5^\circ$ using equation (S.20) from the supporting information.

Excluding the outliers, which result from constraints on the LCModel fit, the *adjusted* ${R}^{2}$ and AICc values for the model described by equation (2) were calculated as 0.86 and −93, respectively; whereas a linear model gave *adjusted* ${R}^{2}$ and AICc values of 0.03 and −71. Hence, the likelihood of equation (2) being the correct model calculated to 99.96%, which is 2436.6 times the likelihood of the linear model.

### Quantification

**Figure 6:**
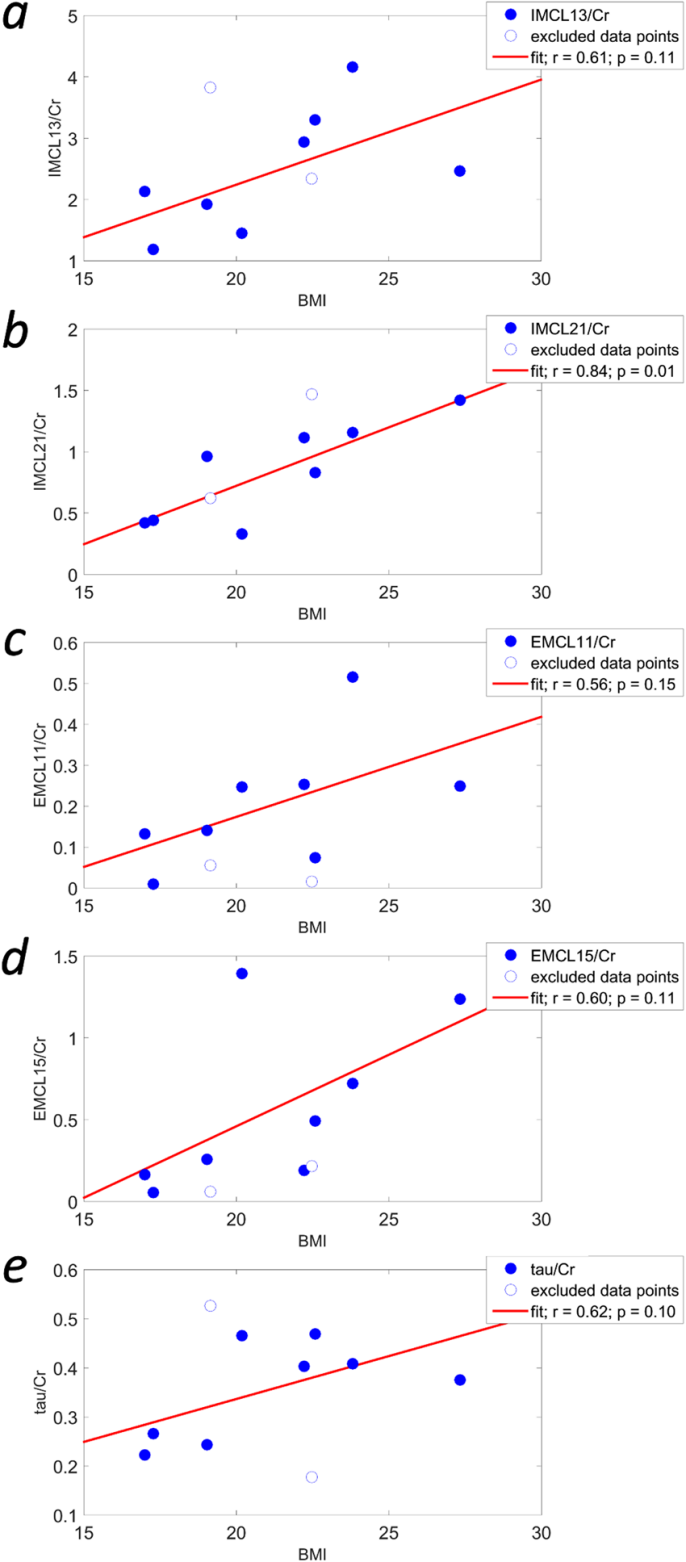
Correlations of metabolite/Cr ratios with BMI. IMCL13/Cr (**a**), IMCL21/Cr (**b**), EMCL11/Cr (**c**), EMCL15/Cr (**d**), and tau/Cr (**e**) plotted versus the body mass index (BMI) of the healthy volunteers. Data points that were excluded from analysis are shown as open circles. Linear regressions are displayed as red lines, and Pearson regression coefficients, *r*, and significance values, *p*, are given for each data set. It can be seen (**b**) that the IMCL21/Cr ratio correlates significantly with BMI. Although not statistically significant, the other metabolite/Cr ratios shown here do exhibit a strong positive correlation with BMI, suggesting that not only unsaturated bonds in IMCL fatty acids (IMCL21) are increased at high BMIs, but also the number of lipid molecules in EMCL, the number of saturated bonds in fatty acids of both compartments, as well as taurine. Other metabolite/Cr ratios that do not show a strong correlation with BMI can be found in the supporting information (Fig. S5.1).

In Fig. 6 the Cr ratios of IMCL13 (a), IMCL21 (b), EMCL11 (c), EMCL15 (d), and tau (e), as given by LCModel, are plotted versus the BMI of the volunteers. It can be seen in Fig. 6(b) that the IMCL21 amplitude correlates significantly with the volunteers’ BMIs ($p < 0.05$). The signal at 2.1 ppm stems from methylene protons adjacent to unsaturated bonds in fatty acids in IMCL. Hence, it appears that a larger BMI leads to a greater concentration of IMCL unsaturated fatty acids in the myocardium. Furthermore, the other lipid moieties shown in the Fig. (a,c,d), and taurine (e), also exhibit a strong positive correlation with BMI that is not statistically significant, likely due to the small sample size. Since the EMCL11 signal stems from the end groups of the lipid chains, this suggests a greater number of lipid molecules in extramyocellular fat, and more saturated bonds in fatty acids in both compartments (IMCL13 and EMCL15), with larger BMIs. Moreover, greater BMI seems to be linked to an increase in the less abundant metabolite taurine. Quantification results of other metabolites without strong correlations to BMI can be seen in the supporting information (Fig. S5.1).

### Prior Knowledge for Quantification

**Figure 7:**
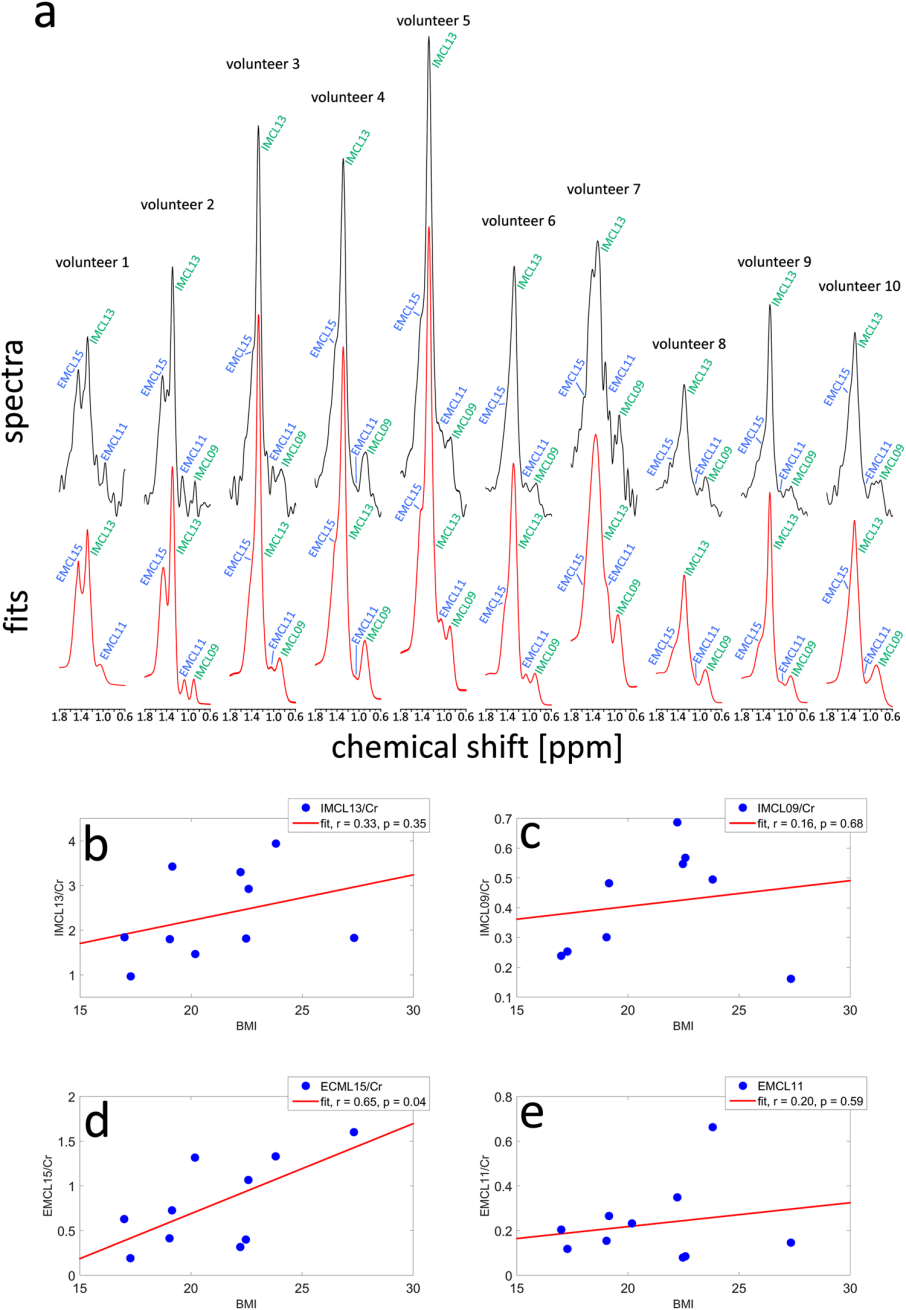
New quantification from LCModel fits incorporating prior knowledge about the chemical shift of lipid resonances. (**a**) Spectra from all ten volunteers in the range from 0.6 ppm to 1.8 ppm are shown (upper panel) together with the fit curves (lower panel) derived from LCModel using the undocumented’chsimu’ parameter to fix the chemical shifts of the most prominent lipid components with respect to each other to the frequencies calculated by equations (3) to (5). Spectra and fits were scaled to match the intensity of the Cr peaks (not shown). While in some cases the EMCL resonances are clearly visible and separated from the IMCL resonances, in other cases the EMCL resonance merges into the IMCL peaks and separation becomes more difficult. Quantification results for IMCL13/Cr (**b**), IMCL09/Cr (**c**), EMCL15/Cr (**d**), and EMCL11/Cr (**e**) from these fits were plotted against the volunteers’ BMIs. Linear regressions are displayed as red lines, and Pearson’s correlation coefficients, *r*, and significance values, *p*, are given for each. It can be seen that the EMCL15/Cr ratio exhibits a significant correlation ($p=0.04$) with the volunteers’ BMIs. Note that, in this case, all data points are included in the analysis, since the inability of the standard LCModel algorithm to reliably quantify EMCL moieties if ${\rm{\Delta }}{\omega }_{EMCL}$ is too small, was corrected in this case by using the ‘chsimu’ parameter, which allowed fixing of the chemical shifts of the lipid components to frequency values calculated with equations (3), (4), and (5).

Figure 7a shows the spectra of all 10 volunteers in the frequency range from 0.6 ppm to 1.8 ppm, which includes the four most prominent lipid moieties: IMCL13, EMCL15, IMCL09, and EMCL11. Also shown is the LCModel fit using prior knowledge about chemical shifts of EMCL, calculated according to equations (3), (4) and (5). Spectra and fits were scaled to the Cr peak. The EMCL components are clearly distinguishable by eye in some cases, but merge with the IMCL peaks in others. Table 2 displays the quantification results of these prior knowledge fits. Comparing the CRLBs of the prior knowledge fits of the lipid moieties here to those of the fits without prior knowledge, the overall CRLBs of IMCL moieties appear to increase only marginally, but decrease substantially for the EMCL components. This indicates that employing prior knowledge of the chemical shift of EMCL components, obtained through calculations from the voxel angle, increases the robustness of quantification results.

**Table 2:** Metabolite/Cr ratios and Cramér-Rao-Lower-Bounds (CRLB) from LCModel with chemical shift of lipid components entered as prior knowledge.

volunteer	IMCL13		EMCL15		IMCL09		EMCL11	
	Cr ratio	CRLB [%]	Cr ratio	CRLB [%]	Cr ratio	CRLB [%]	Cr ratio	CRLB [%]
1	1.47	5	1.32	5	—	—	0.23	19
2	1.82	4	1.60	4	0.16	20	0.15	23
3	3.43	2	0.73	9	0.48	8	0.27	13
4	2.92	2	1.06	5	0.57	4	0.08	19
5	3.94	1	1.33	4	0.49	6	0.66	6
6	1.83	3	0.63	8	0.24	10	0.21	13
7	3.30	2	0.32	21	0.69	9	0.35	14
8	0.97	4	0.19	14	0.25	9	0.12	24
9	1.80	2	0.42	6	0.30	7	0.15	12
10	1.81	4	0.40	17	0.55	9	0.08	42
**Mean (SD)**	2.33 (0.94)	2.90 (1.22)	0.80 (0.47)	9.3 (5.69)	0.24 (0.17)	9.11 (4.23)	0.23 (0.17)	18.50 (9.39)

Cr ratios and CRLBs of the four most prominent lipid moieties are given after re-fitting the spectra with LCModel, this time using prior knowledge regarding the angular dependence of the chemical shift of EMCL. To this end, the undocumented ‘chsimu’ parameter was employed, which allowed fixing of the chemical shifts of these lipid components to frequency values calculated from equations (3), (4), and (5). Means and standard deviations (SD) over all volunteers are given in the bottom row. Compared to the fit without prior knowledge, the CRLBs of IMCL components appear to increase only marginally, while CRLBs of EMCL components decrease substantially in most cases.

In Fig. 7b–e the quantification results for the four lipid components are plotted versus BMI. The EMCL15/Cr ratios obtained from the prior knowledge fits correlate significantly ($p < 0.05$) with the volunteers’ BMIs (Fig. 7d). Note that in this case all data points are included in the analysis.

## Discussion

We have demonstrated that the newly-introduced IVS-McPRESS technique is capable of very high spectral data quality, which likely enables the separation and isolated analysis of IMCL and EMCL components in the myocardium for the first time, as well as the analysis of less abundant metabolites, such as trimethylamine (TMA) and taurine (tau). This is possible because very narrow linewidths were achieved. Unfortunately, many publications fail to report the average water peak linewidths of their spectra, making a proper comparison difficult. Positive exceptions are Bottomley *et al*.^41^, Machann *et al*.^42^ and Rial *et al*.^25^, who reported mean (SD) linewidths of 19 (8) Hz, 13 to 17 Hz, and 13.3 (2.4) Hz at 3 T, respectively. Although the linewidth reported in the last of these publications is comparable to the mean (SD) water peak linewidth of 12 (1) Hz shown in our study, it should be noted that Rial *et al*.^25^ extracted the linewidth from spectra of only four signal averages acquired in a single breath-hold, thus avoiding the most important line-broadening issues like respiratory motion and thereby introduced field fluctuations. Such an approach, however, compromises the spectral SNR, making reliable metabolite quantification challenging, particularly for less abundant metabolites. Weiss *et al*.^43^ and Van der Meer *et al*.^5^ reported average water peak linewidths of 8.6 Hz (1.5 Hz) and 10.7 Hz (0.4 Hz) at 1.5 T, respectively; however, at this field strength, linewidths are expected to be approximately half the widths of those observed at 3 T. The narrow linewidth at 3 T in this study was achieved via: (1) a small voxel size; (2) the use of IVS for robust localisation to the interventricular septum; (3) a sophisticated image-based B_0_ shimming strategy; and (4) a non-water suppressed MRS sequence in conjunction with ECG triggering and navigator respiratory-motion tracking, which allowed for prospective and retrospective motion correction, as well as frequency alignment and phase correction during post-processing. Moreover, the measurements shown here were acquired on a 3 T system, while most ^1^H CMRS studies so far were performed at 1.5T^1^. The increased field strength results in a higher SNR and thus counteracts SNR loss resulting from the small voxel size. Furthermore, it leads to an increased frequency shift of the EMCL resonances with respect to the IMCL frequencies, facilitating more reliable separation of both signals.

Our investigation of the chemical shift difference between EMCL15 and IMCL13, ${\rm{\Delta }}{\omega }_{EMCL}$, and its dependence on the angle the voxel takes with the static magnetic field B_0_, proves the feasibility of reliably separating signals from IMCL and EMCL within the human myocardium with the proposed technique. However, we also demonstrate that robust separation of signals from the different lipid compartments is only reliably possible if the mean angle, $\theta$, of the myocardial muscle fibres with B_0_ in the voxel of interest is between $-24.5^\circ  < \theta  < 24.5^\circ$, the range where ${\rm{\Delta }}{\omega }_{EMCL}$ is large enough for the two signals from the different compartments to be resolved. Furthermore, we demonstrated that employing prior knowledge about the chemical shift of the EMCL signals improves robustness and reliability of quantification results. This prior knowledge can be derived from the voxel orientation with respect to B_0_, if certain conditions are met, like consistent voxel positioning with respect to myofibre orientation. However, at angles $\alpha$ where the resonances of the two lipid moieties coincide at the same frequency, separation of signals from the two lipid compartments is impossible. Hence, care must be taken during the positioning and angulation of the examination voxel along the curved surface of the interventricular septum. It is worth pointing out that there is a smooth transition of myocardial fibre orientations from endocardium to epicardium through the septum^44–46^; however, the peak position of the EMCL signal will correspond to the mean fibre orientation, with the linewidth depending on the distribution of fibre orientations within the voxel (see the supporting information for further details). Nevertheless, data from this study are in excellent accordance with both the findings for skeletal muscle regarding the dependence of the chemical shift of EMCL signals on fibre orientation reported by Boesch *et al*.^9^ and the theoretical considerations laid out in the appendix of the same reference. Our findings add to this body of literature, and will permit improved lipid quantification in skeletal muscle as well as myocardium. However, the findings of this study are based solely on examinations in healthy volunteers, and need to be evaluated in pathological conditions where myocardial fibre structure is altered, such as myocardial infarction^47^, which leads to structural remodelling.

In this study Cr39 was excluded from more detailed analysis, as its CRLBs calculated by LCModel were high, and quantification was thus unreliable. The poor quality of this resonance could result from signal loss caused by imperfect inversion by the MC pulse, due to its proximity to the water resonance. Furthermore, the frequency fluctuations between individual signal acquisitions (shown in Fig. S3.1 in the supporting information) by cardiac and respiratory motion caused partial inversion of the water resonance, to varying extents in different acquisitions. This, and the heartrate dependent TR, led to spectra with fully-relaxed metabolite signals, but only partly – and inconsistently over different volunteers – relaxed water signals made water an unreliable quantification reference in this study (T_1_ values of methylene groups and water in skeletal muscle are <0.5 s and approximately 1.4 s, respectively^48^). Hence, for water referencing a non-water-suppressed acquisition with sufficient TR and without metabolite cycling will be required in future CMRS work, although metabolite cycling theoretically offers the option to calculate a pure water signal without an additional scan. Clearly, further improvement of quantification will require a more rigorous investigation of relaxation rates of different spectral components in cardiac muscle.

Due to the lack of standardised analysis tools for ^1^H CMRS, the analysis and quantification of ^1^H spectra acquired from the myocardium remains challenging. In a first approach to analyse the spectral data acquired from the hearts of volunteers, it was assumed that the heart is a muscle and its chemical composition would be similar to that of skeletal muscle. Hence, the spectral basis sets of a skeletal muscle model in the standard spectroscopy analysis tool LCModel were presumed to suffice for approximation of the measured metabolite concentrations. This assumption, however, holds true only to a certain point.

We demonstrated in this study that it is possible to resolve and individually quantify the IMCL13 and EMCL15 signals by accounting for their chemical shift difference and its dependence on the cardiac fibre angle. However, based on the LCModel results it was not possible to establish the same relationship for the less-pronounced IMCL and EMCL moieties. This failure can be partly explained by the low signal amplitudes of the respective resonances, which make reliable peak fitting difficult, especially in the presence of other signals overlapping the resonance in question. Furthermore, it has been shown in previous studies^13–16^ that the MR spectrum of triglycerides, in the absence of any susceptibility effects due to muscular structure, exhibits a resonance peak from CH_2_ groups next to the glycerol resonance at 2.3 ppm. Therefore, the signal fitted with a single basis function at 2.3 ppm reflects the sum of the IMCL signal at that frequency and EMCL signal, which corresponds to the IMCL21 signal but is shifted due to susceptibility effects. Hence, the chemical shift difference between the EMCL23 signal and the IMCL21 signal cannot be determined from the LCModel fit. Moreover, the basis functions used to fit IMCL21 and the combined I/E23 signal contain a second very broad resonance upfield to the main peak of these basis functions (Fig. 4). The two components of these basis functions were introduced in a previous study to approximate lipid contributions that were not well-specified and could not be further resolved in the spectra acquired in that study, presumably due to the low static field applied. However, the data of this study show that these broad features do not capture the different spectral components of the lipids in the spectra very well, and instead result in overfitting of the data and a visible dip in the residual. Two additional resonances in the triglyceride spectrum are not considered by the LCModel fit: one at 1.6 ppm and another at 2.75 ppm, originating from the methyl protons next-nearest to the glycerol and from polyunsaturated fatty acids, respectively. These resonances were likely difficult to resolve *in vivo*, due to low signal amplitudes, and were therefore neglected in the underlying model. Nevertheless, by summing the residuals of the acquired spectra from this study (again excluding the spectra where EMCL15 was not correctly fit), a low-amplitude resonance peak became apparent at about 2.7 ppm (see Fig. S4.3). It is clear from these considerations that ^1^H CMRS quantification could be greatly improved by incorporating the extensive prior knowledge regarding the complex spectral structure of the triglyceride spectrum into the models used for peak fitting procedures, namely: (1) the known structure of the triglyceride spectrum with all relevant resonances^13,14^; (2) the correlations of different peak amplitudes to one another within the triglyceride spectrum to better estimate concentrations of overlapping peaks as demonstrated by Hamilton *et al*.^16^; (3) the knowledge that the chemical shift difference between EMCL and IMCL signals is a result of susceptibility effects, and is therefore equal between all IMCL resonances and the corresponding EMCL resonances^9^; and (4) the dependence of the chemical shift difference on the angle $\theta$ between cardiac fibres and the main magnetic field B_0_, as described by equation (2), as well as the voxel angle $\alpha$, after a proper relationship between $\alpha$ and $\theta$ has been established. An early indication of the expected improvement in such measures was given in this study: fitting the data a second time, and fixing the chemical shifts of the four most prominent lipid moieties according to the values calculated by equations (3) to (5), led to a substantial decrease in the CRLBs of the EMCL signals and a statistically significant correlation between the EMCL15/Cr ratio and the volunteers’ BMIs was found. Hence, developing and verifying a quantification model capable of capturing the complexity of triglycerides in muscle and cardiac muscle spectra will be a challenging but worthy future endeavour.

The improved spectral quality achievable with the IVS-McPRESS method shown here, together with more sophisticated quantification routines as outlined above, may allow deeper insight into cardiac metabolism in healthy subjects and diverse patient groups. In particular, our data show a strongly significant correlation between the IMCL21/Cr ratio ($p=0.01$, $r=0.84$), and the EMCL15/Cr ratio ($p=0.04$, $r=0.65$) and the volunteers’ BMIs, suggesting that a higher BMI is linked to a greater concentration of intramyocellular unsaturated fatty acids as well as extramyocellular saturated fatty acids. Furthermore, the number of extracellular triglyceride molecules, as well as the number of saturated bonds in intramyocellular fatty acids, appears to increase with increasing BMI; the signals from EMCL11/Cr ratios, and IMCL13/Cr ratios, namely the number of end groups in EMCL and the number of methylene groups in IMCL, respectively, exhibit strong correlations with BMI ($r=0.56$, and $r=0.61$ respectively), although these are not statistically significant. Another strong but non-statistically-significant correlation was found between BMI and taurine/Cr ratios ($r=0.62$). While a linear correlation seems plausible between increasing BMI and lipid levels from both compartments, further studies on larger samples are required to confirm these changes and establish their statistical significance and clinical relevance. Moreover, further analysis on a broader database may reveal more complex relationships between other lipid moieties and metabolite concentrations and BMI that cannot be seen in this study, due to the small sample size.

## Conclusions

For the first time, the feasibility of IVS-McPRESS, a metabolite-cycled non-water-suppressed ^1^H CMRS technique, to improve spectral quality in ^1^H MRS in the human myocardium was demonstrated, and the data suggest that separation and individual analysis of IMCL and EMCL signals in the myocardium is possible. Prior knowledge of the angular dependence of the chemical shift of EMCL signals was used to confirm quantification results and their assignment to the different lipid compartments, and strong correlations between concentrations of different lipid moieties and volunteers’ BMIs were found. These results indicate that it is possible to reliably resolve signals from different lipid compartments at 3 T, provided the myofibre orientation within the voxel of interest with the main magnetic field is between $-24.5^\circ  < \theta  < 24.5^\circ$. This will enable improved ^1^H MRS quantification accuracy in studies of myocardial metabolism, and will also prove useful in skeletal muscle applications.

### Declarations

#### Ethics approval and consent to participate

This study was approved by the cantonal ethics board of Zurich under the reference number EK: 09/2006(ETH). All volunteers gave written informed consent for participation.

#### Consent for publication

Written informed consent was obtained from the participants for publication of their individual details. The consent form is held by the authors’ institution and is available for review by the Editor-in-Chief.

#### Availability of data and material

The datasets generated, used and analysed during the current study are available from the corresponding author on reasonable request.

## Electronic supplementary material

Supporting Information
