# Supplementary material for: Non-Water-Suppressed 1H MR Spectroscopy with Orientational Prior Knowledge Shows Potential for Separating Intra- and Extramyocellular Lipid Signals in Human Myocardium
Source: Sci Rep. 2017 Dec 4;7:16898. doi: 10.1038/s41598-017-16318-0 (PMC5714998; doi:10.1038/s41598-017-16318-0)
Supplement: Supplementary file 1 — Supporting Information [file 41598_2017_16318_MOESM1_ESM.pdf]

Supporting Information:

Non-Water-Suppressed ^{1}H MR Spectroscopy with Orientational Prior Knowledge for Robust Separation of Intra- and Extramyocellular Lipid Signals in Human Myocardium

A Fillmer, A Hock, D Cameron, and A Henning

1. Bulk susceptibility effects cause a frequency shift of the EMCL-signal in muscle tissue

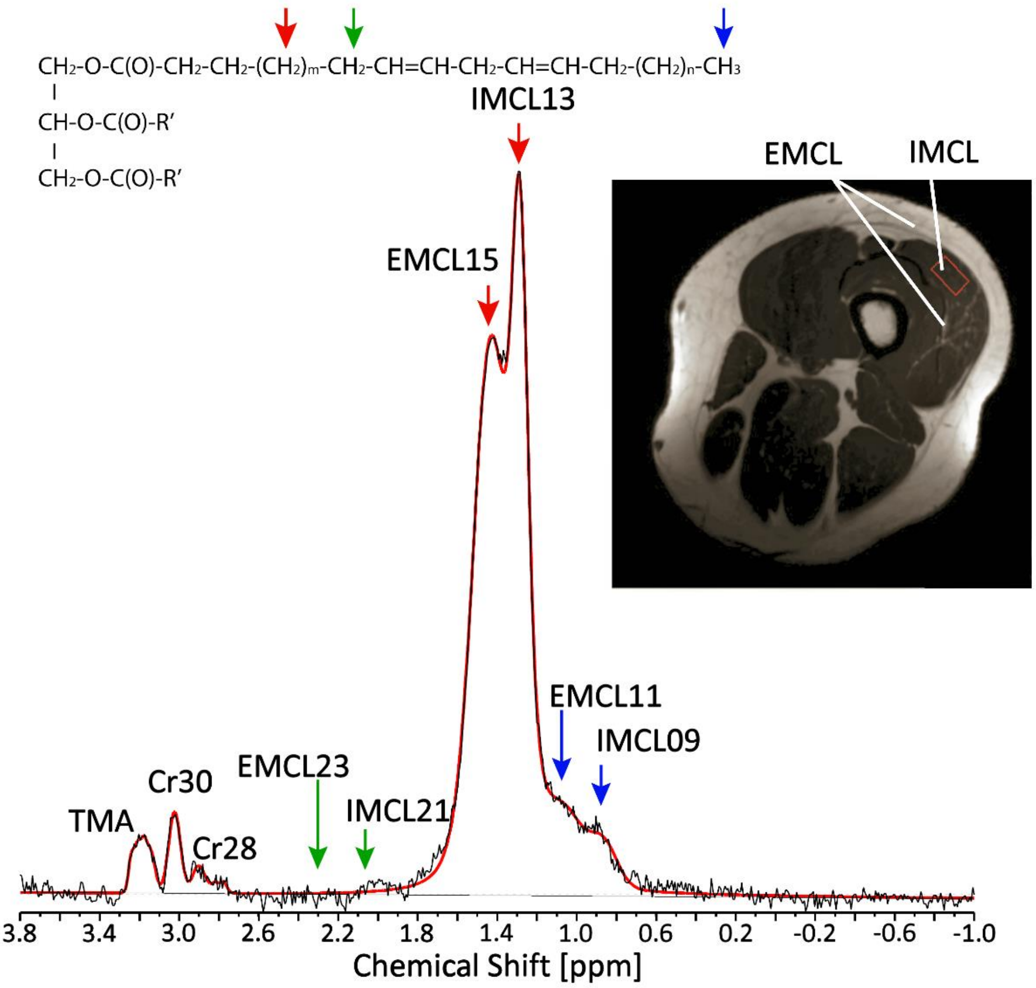

***Figure S1.1: Representative ^{1}H MR spectrum from the vastus lateralis muscle**. The voxel position for this measurement is indicated in the inlay on the right. The intramyocellular lipid (IMCL) signal stems from lipids within muscle cells, which appear as dark regions as indicated in the inlay image. The extramyocellular lipid (EMCL) signal stems from lipids outside of muscle cells. Larger areas of lipids outside of the muscle cells, like subcutaneous fat or fasciae between different muscles, appear as bright regions, as indicated in the inlay. However, EMCL occurs also in small quantities between muscle cells. These EMCL compartments cannot be visualized by MR imaging. The splitting of the IMCL and EMCL resonances in the spectrum is clearly visible. The linewidth of the unsuppressed water peak of this measurement is 16 Hz. At the top of the figure the structural formula of a lipid molecule is displayed, with the relevant ^{1}H positions and their respective resonance peaks being indicated by the red, green and blue arrows.*

2. Relationship between voxel orientation and myocardial fibre orientation and their influence on the chemical shift of EMCL

Previous studies have shown that the chemical shift of extramyocellular lipids (EMCL) relative to intramyocellular lipids (IMCL), $\Delta\omega_{EMCL}$, depends on the angle, θ, muscle fibres take with the orientation of the main magnetic field B_0. As illustrated in Figure S2.1, the angles θ and $\theta + \pi$ are equivalent, and these are also equivalent to the angles – θ and $-\theta - \pi$, as a rotation around B_0 does not change the angle the muscle fibres take with B_0. Therefore, $\Delta\omega_{EMCL}$ can be described by an even function with a period of π, such as a $\cos^2$ function. Boesch et al. have calculated the minimum and maximum chemical shift of the EMCL resonances relative to their corresponding IMCL resonances to be 0.21 ppm, if the muscle fibres are oriented parallel to the main magnetic field, and -0.1 ppm, if the muscle fibres are orthogonal to the main magnetic field[1]. Hence, $\Delta\omega_{EMCL}$ can be calculated in terms of θ as follows:

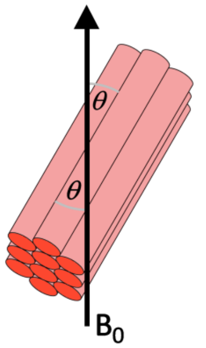

Figure S2.1: Since muscle fibres have an orientation, but not a direction in the sense of being a vector, they cannot be oriented antiparallel to the main magnetic field B_0. Hence, if the muscle fibres take an angle $\boldsymbol{\theta}$ with B_0, they simultaneously take an angle $\boldsymbol{\theta + \pi}$ with B_0. Furthermore, a rotation around B_0 does not change the angle between B_0 and the muscle fibres. Hence, the four angles $\boldsymbol{\theta}$, $\boldsymbol{\theta + \pi}$, $\boldsymbol{-\theta}$, and $\boldsymbol{-\theta + \pi}$ are equivalent.

(S.1) $$\Delta\omega_{EMCL}(\theta) = 0.31 \cdot \cos^2(\theta) - 0.1.$$

However, in the heart, the orientation of muscle fibres within the interventricular septum relative to B_0 is usually not exactly known, and likely shows inter-individual variation.

Assuming it is possible to consistently position the spectroscopy voxel with respect to the myocardial fibre orientation, the angle the voxel takes with B_0, α, will have a fixed, yet unknown, relationship with θ. Thus, θ can be expressed as a function of α:

(S.2) $$\theta = f(\alpha).$$

To derive a general form of $f(\alpha)$, some simple assumptions are required. First, let $\vec{a}$ be a vector that indicates the voxel orientation. Given that the length of the vector is not important and only the orientation is of interest, we can assume the following:

(S.3) $$|\vec{a}| = 1.$$

Second, we assume the voxel is oriented parallel to the external magnetic field, B_0, which is conventionally parallel to the z-axis and can thus be expressed by the unit vector $\vec{z}$:

(S.4) $$\vec{a} = \vec{z} = \begin{pmatrix} 0 \\ 0 \\ 1 \end{pmatrix},$$

which leads to

(S.5) $$\alpha = 0.$$

Similarly, we can define another vector, $\vec{b}$, that is parallel to the cardiac fibre orientation:

(S.6) $$|\vec{b}| = 1.$$

Assuming the voxel can be positioned and oriented reproducibly relative to the muscle fibres, the voxel orientation, $\vec{a}$, and the cardiac fibre orientation, $\vec{b}$, will take a fixed angle with each other, which we call ρ.

For simplicity, we assume $\vec{b}$ lies in the x-z plane for now. This means $\vec{b}$ can be calculated by applying a rotation matrix $\mathbf{R}_y$ to $\vec{z}$, which rotates $\vec{z}$ around the y-axis by the angle ρ:

$$\text{(S.7)} \quad \vec{b} = \mathbf{R}_y(\rho)\vec{z} = \begin{pmatrix} \cos(\rho) & 0 & \sin(\rho) \\ 0 & 1 & 0 \\ -\sin(\rho) & 0 & \cos(\rho) \end{pmatrix} \cdot \vec{z}.$$

The angle θ between the muscle fibre orientation, $\vec{b}$, and B_0 can then be calculated by:

$$\text{(S.8)} \quad \cos(\theta) = \frac{\vec{z} \cdot \vec{b}}{|\vec{z}| \cdot |\vec{b}|}.$$

Clearly, when the voxel orientation $\vec{a}$ is parallel to B_0, θ equals ρ.

Now let us assume the voxel orientation $\vec{a}$ is not parallel to B_0, but is rotated by an angle $\alpha \neq 0$. For the sake of simplicity, we define the axis of rotation to be the x-axis. The new voxel orientation can then be calculated as:

$$\text{(S.9)} \quad \vec{a}' = \mathbf{R}_x(\alpha)\vec{a} = \begin{pmatrix} 1 & 0 & 0 \\ 0 & \cos(\alpha) & -\sin(\alpha) \\ 0 & \sin(\alpha) & \cos(\alpha) \end{pmatrix} \cdot \vec{a}.$$

Again, assuming a fixed voxel orientation relative to the cardiac fibre orientation, we can calculate the cardiac fibre orientation in this rotated scenario by a rotation around the x-axis by the angle α:

$$\text{(S.10)} \quad \vec{b}' = \mathbf{R}_x(\alpha)\vec{b} = \begin{pmatrix} 1 & 0 & 0 \\ 0 & \cos(\alpha) & -\sin(\alpha) \\ 0 & \sin(\alpha) & \cos(\alpha) \end{pmatrix} \cdot \vec{b}.$$

Calculating θ according to equation S.8 for different angles α, and substituting $\vec{b}'$ for $\vec{b}$, it becomes

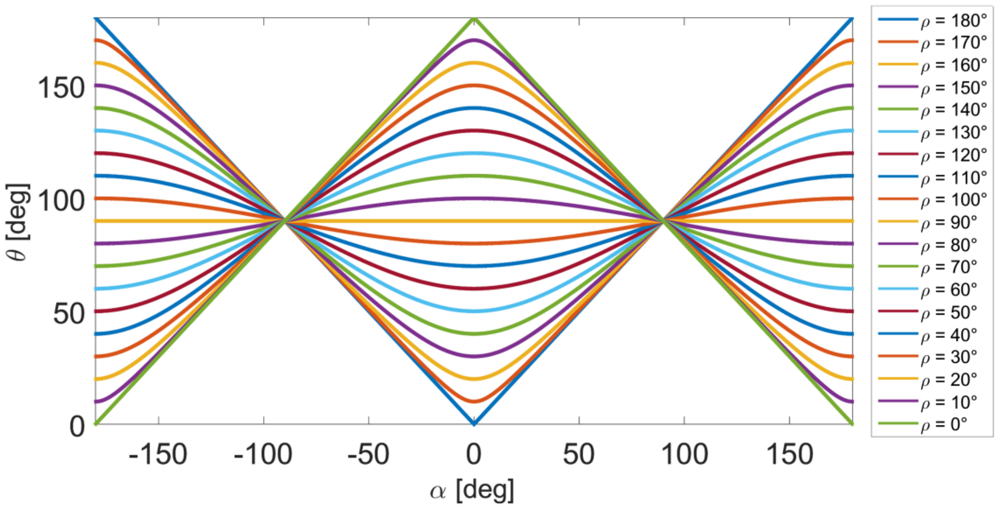

Figure S2.2: The angle θ the vector $\vec{b}'$ (vector $\vec{b}$ after rotation) takes with the main magnetic field, B_0, if vectors $\vec{a}$ and $\vec{b}$, which are parallel to the voxel orientation and the hypothetical fibre orientation, respectively, are rotated around the x-axis by an angle α. For $\alpha = 0°$, $\vec{a}$ is defined to be oriented parallel to B_0 and the z-axis; therefore, $\vec{a}'$, the vector $\vec{a}$ after rotation, will always take the angle α with the z-axis. The different colours represent different angles ρ, which vectors $\vec{a}$ and $\vec{b}$ take with each other. During the rotation the angle ρ remains constant. Clearly, while α is swept through all values between -180° and +180°, the range of the angle θ is compressed, depending on the angle ρ.

clear that while α can be swept through all values from -180° to +180°, the range of values θ can take is smaller, unless $\rho = 0$. The link between α and θ is illustrated in Figure S2.2 for different angles ρ.

If we use equation S.1 to calculate $\Delta\omega_{\mathrm{EMCL}}$ in terms of θ, which is restricted in its range, this leads to the range of $\Delta\omega_{\mathrm{EMCL}}$ being compressed as well, as shown in Figure S2.3.

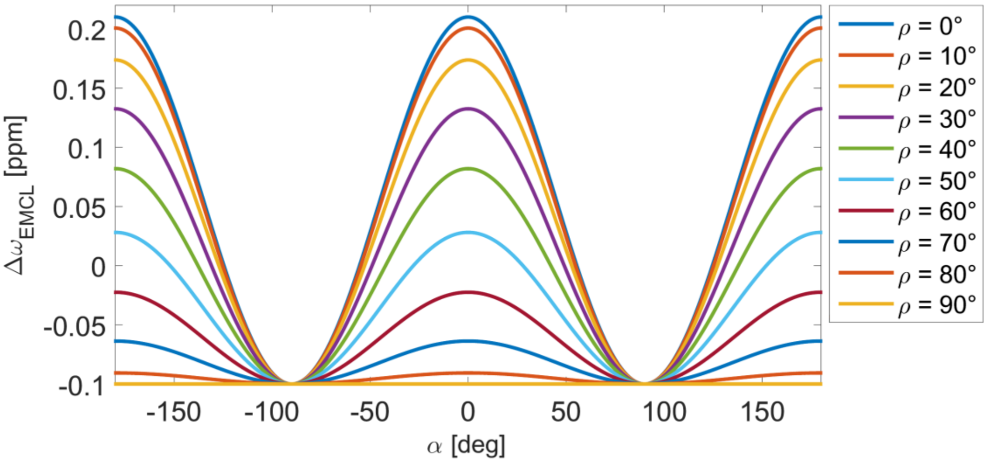

Figure S2.3: The chemical shift difference of EMCL, $\Delta\omega_{\mathrm{EMCL}}$, compared to the fixed chemical shift of IMCL, calculated according to equation (S.1) for different angles θ of the fibre orientation with respect to B_0. Angle θ was calculated from equations (S.7), (S.8), and (S.10), and is hence dependent on α and ρ. The different colours represent different angles ρ between the voxel orientation and the fibre orientation. It can be seen that the range of $\Delta\omega_{\mathrm{EMCL}}$ is dependent on the angle ρ.

From Figure S2.3 it is clear that the compression of the range of $\Delta\omega_{\mathrm{EMCL}}$ depends on the angle ρ the voxel takes with the fibre orientation. To explore this dependency further, we calculated the scaling factor:

$$\text{(S.11)} \quad \mathrm{f_{sc}} = \frac{\Delta\omega_{\mathrm{EMCL}}(\rho)}{\Delta\omega_{\mathrm{EMCL}}(\rho=0)},$$

and plotted it against ρ, as shown in Figure S2.4. From this, the following relationship becomes apparent:

$$\text{(S.12)} \quad \mathrm{f_{sc}} = \cos^2(\rho).$$

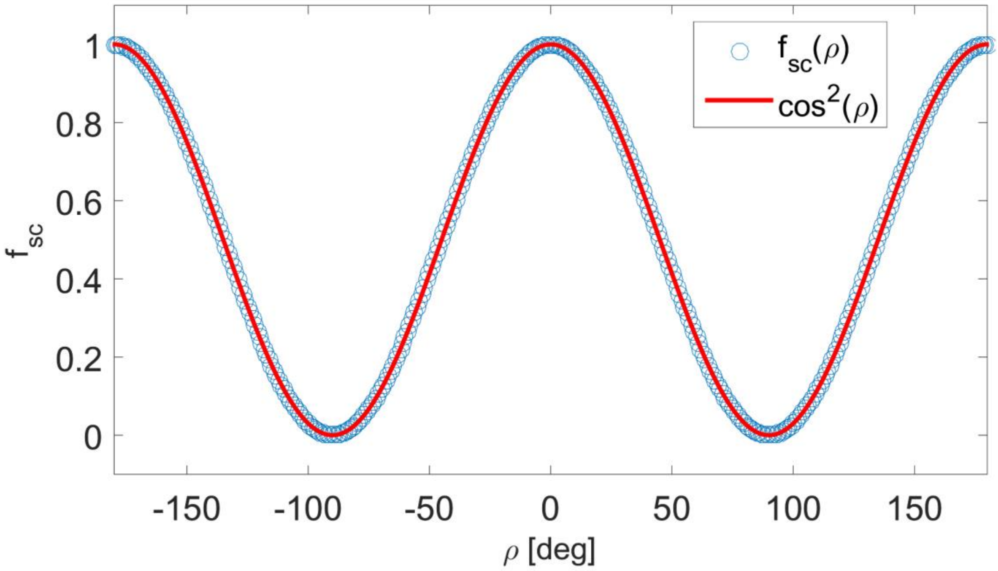

Figure S2.4: The scaling factor $\mathrm{f_{sc}}$ of the range of $\Delta\omega_{\mathrm{EMCL}}$ depending on the angle ρ between the fibre orientation and the voxel orientation. It can be seen that $\mathrm{f_{sc}} = \cos^2(\rho)$.

Hence, as long as the vectors defining the voxel orientation, $\vec{a}$, the fibre orientation, $\vec{b}$, and the rotation axis, in this example the x-axis, are all defined in one plane, we can rewrite equation (S.1) as:

$$\text{(S.13)} \quad \Delta\omega_{\mathrm{EMCL}}(\alpha) = \cos^2(\rho) \cdot 0.31 \cdot \cos^2(\alpha) - 0.1,$$

with

$$\text{(S.14)} \quad \cos^2(\theta) = \cos^2(\rho)\cos^2(\alpha),$$

or

$$\text{(S.15)} \quad \theta = f(\alpha) = \arccos\left(\sqrt{\cos^2(\rho)\cos^2(\alpha)}\right).$$

However, in reality the voxel orientation, the fibre orientation, and the rotation axis do not necessarily lie within the same plane. To account for this in the equations above, an additional rotation around the x-axis by an angle α_θ can be applied to $\vec{b}$, the fibre orientation, so it is no longer in the x-z-plane:

$$\text{(S.16)} \quad \vec{b} = \mathbf{R}_x(\alpha_\theta)\mathbf{R}_y(\rho)\vec{z} = \begin{pmatrix} 1 & 0 & 0 \\ 0 & \cos(\alpha_\theta) & -\sin(\alpha_\theta) \\ 0 & \sin(\alpha_\theta) & \cos(\alpha_\theta) \end{pmatrix} \begin{pmatrix} \cos(\rho) & 0 & \sin(\rho) \\ 0 & 1 & 0 \\ -\sin(\rho) & 0 & \cos(\rho) \end{pmatrix} \cdot \vec{z}.$$

As described above, the angle θ can be calculated using equation S.8. Clearly, with the addition of α_θ, θ will no longer equal ρ, unless $\alpha_\theta = 0$. Furthermore, the angle the voxel takes with the fibres, which is unknown but assumed to be constant, is no longer equal to ρ, as in the scenario above. We define this angle as follows:

$$\text{(S.17)} \quad \varphi = \arccos\left(\frac{\vec{a} \cdot \vec{b}}{|\vec{a}| \cdot |\vec{b}|}\right).$$

Rotating the whole system around the x-axis, as described above, we can calculate the different angles θ for corresponding angles α and α_θ, as is illustrated in Figure S2.5. It can be seen that applying the additional rotation, α_θ, around the x-axis does not change the range of values θ can assume, but shifts the function $\theta(\alpha)$ by $-\alpha_\theta$.

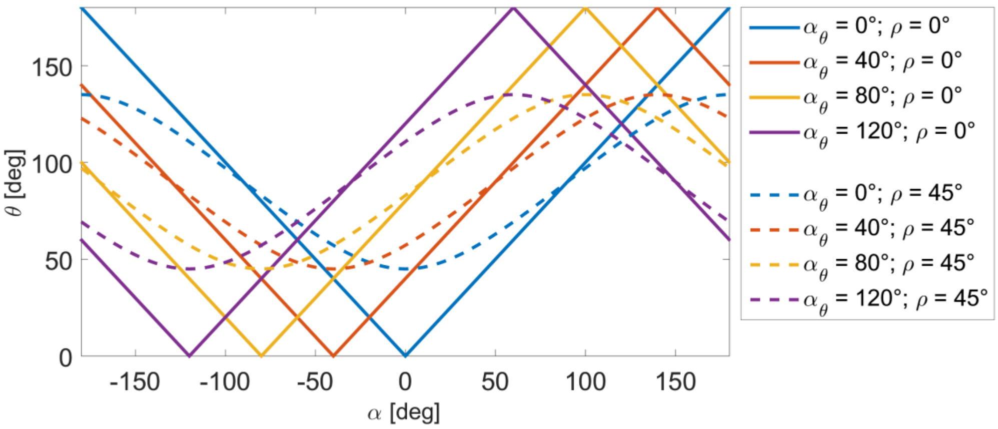

Figure S2.5: The angle θ versus α, calculated for different angles of α_θ – the angle by which the fibre orientation is tilted out of the plane that is spanned by the voxel orientation and the rotation axis - and ρ. It is clear that the maximum and minimum amplitude of θ is not affected if the voxel orientation is not lying in the same plane as the rotation axis and the voxel orientation; however, the whole function $\theta(\alpha)$ is shifted by $-\alpha_\theta$, the angle by which the fibre orientation is tilted from that plane.

In the same way, $\Delta\omega_{\mathrm{EMCL}}$ is shifted along the horizontal axis, denoting the rotation angle α, by a constant offset of $-\alpha_\theta$, as shown in Figure S2.6.

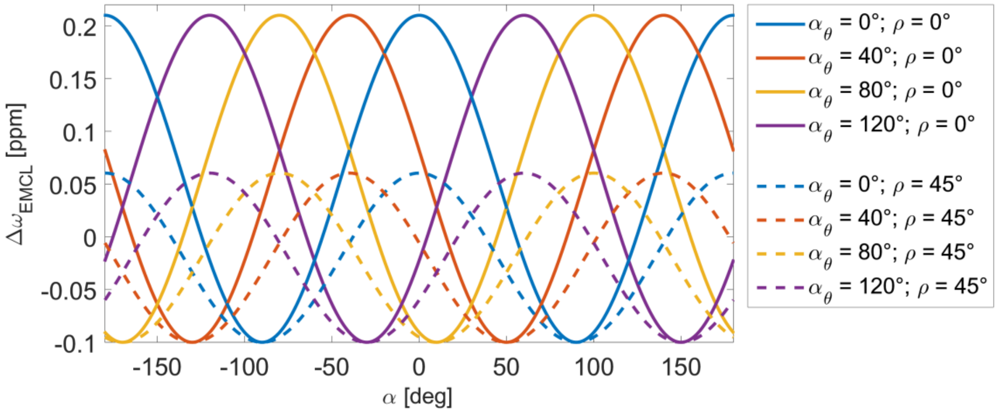

Figure S2.6: The chemical shift difference of EMCL, $\Delta\omega_{\mathrm{EMCL}}(\alpha)$, calculated for different angles α_θ and ρ. It can be seen that the maxima are dependent on the angle ρ, while the "phase", namely the rotation angle α for which maximum and minimum chemical shift differences are observed, is determined by the angle α_θ.

Hence, equation (S.1) can be rewritten more generally as:

$$\text{(S.18)} \quad \Delta\omega_{\mathrm{EMCL}}(\alpha) = \cos^2(\rho) \cdot 0.31 \cdot \cos^2(\alpha - \alpha_\theta) - 0.1,$$

with

$$\text{(S.19)} \quad \cos^2(\theta) = \cos^2(\rho)\cos^2(\alpha - \alpha_\theta),$$

and, finally, θ can be substituted by:

$$\text{(S.20)} \quad \theta = f(\alpha) = \arccos\left(\sqrt{\cos^2(\rho)\cos^2(\alpha - \alpha_\theta)}\right).$$

The use of equation S.18 therefore allows us not only to investigate the angular dependence of the chemical shift difference, $\Delta\omega_{EMCL}$, on the angle α the voxel takes with B_0, but also to estimate the angle θ, the cardiac fibres take with B_0.

While these calculations focus on one main fibre angle, it is known that the fibre angle rotates when moving from epicardium to endocardium[2-4]. However, the fibre angle distribution is not isotropic, and hence a mean fibre orientation exists. This mean fibre orientation will determine the mean chemical shift of the EMCL moieties. Nevertheless, each individual EMCL molecule within the voxel contributes to the acquired signal, and EMCL from adipocites between myofibres with a different angulation will exhibit a different chemical shift from the mean. Hence, the distribution of fibre angulations will lead to a broadening of the EMCL signals.

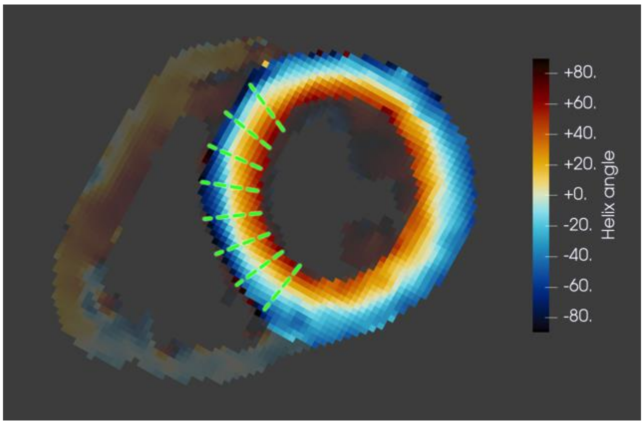

Figure S2.7: Map of helix angles of the myocardial fibres of the left-ventrical wall in a short axis view during systole. This map was derived from a cardiac DTI acquisition. It can be seen that the helix angle in the mesocardium is approximately parallel to the tangent of the according segment of the left ventrical wall. The green lines indicate profiles across which the transition of the helix angle from endocardium to epicardium was calculated, in order to determine a mean distribution of helix angles across different volunteers, as displayed in Figure S2.7. (This image is provided courtesy of Dr. Pedro F. Ferreira, Royal Brompton Hospital, London.)

From diffusion tensor imaging (DTI) of the heart, it can be seen that the mean helix angle in the mesocardium is approximately circumferential with the left ventricle in a short axis view. Figure S2.7 shows the visualisation of such a helix angle distribution across the wall of the left ventricle in a short axis view, derived from a DTI study[2]. From these helix angle maps, helix angle profiles across the septal wall were calculated in 19 volunteers, which were the control cohort in a study published by Nielles-Vallespin et al.[2]. Then a mean profile of helix angles across the septal wall was calculated for each volunteer, and finally a median profile was obtained across all 19 volunteers, which is shown in Figure S2.8. The error bars indicate the inter-subject inter quartile range. It can be seen that the helix angles vary between approximately -60° to +60°. However, in our ^{1}H MRS measurements, inner volume saturation[5] saturates the regions with the most extreme helix angles. Furthermore, it can be seen that the inter-subject variability of helix angles, especially in the mesocardium, is rather small. Hence, it seems justified to assume that it is possible to consistently place the voxel with respect to the myocardial fibre orientation, and to derive information about the myocardial fibre orientation from the voxel angle, using the above outlined calculations. (Data and images about the helix angle distribution across the septal wall are provided courtesy of Dr. Pedro F. Ferreira, Royal Brompton Hospital, London.)

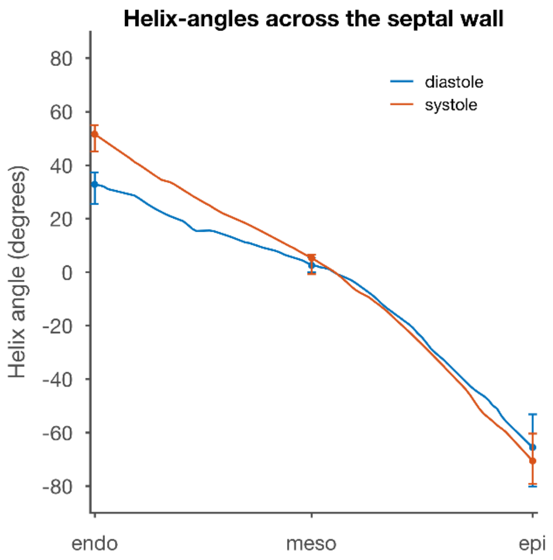

Figure S2.8: Helix angles across the septal wall during systole (orange) and diastole (blue). Helix angle profiles were calculated in 19 healthy volunteers (controls from the study published in (ref)). First, several profiles were calculated in each volunteer (example lines across which the profiles were calculated are indicated as green lines in Figure S2.6), then a mean profile was calculated for each volunteer. Finally, a median profile was calculated across all 19 volunteers. The error bars indicate the inter-subject inter-quartile range. (This image is provided courtesy of Dr. Pedro F. Ferreira, Royal Brompton Hospital, London.)

3. Effects of phase correction and frequency alignment during post-processing

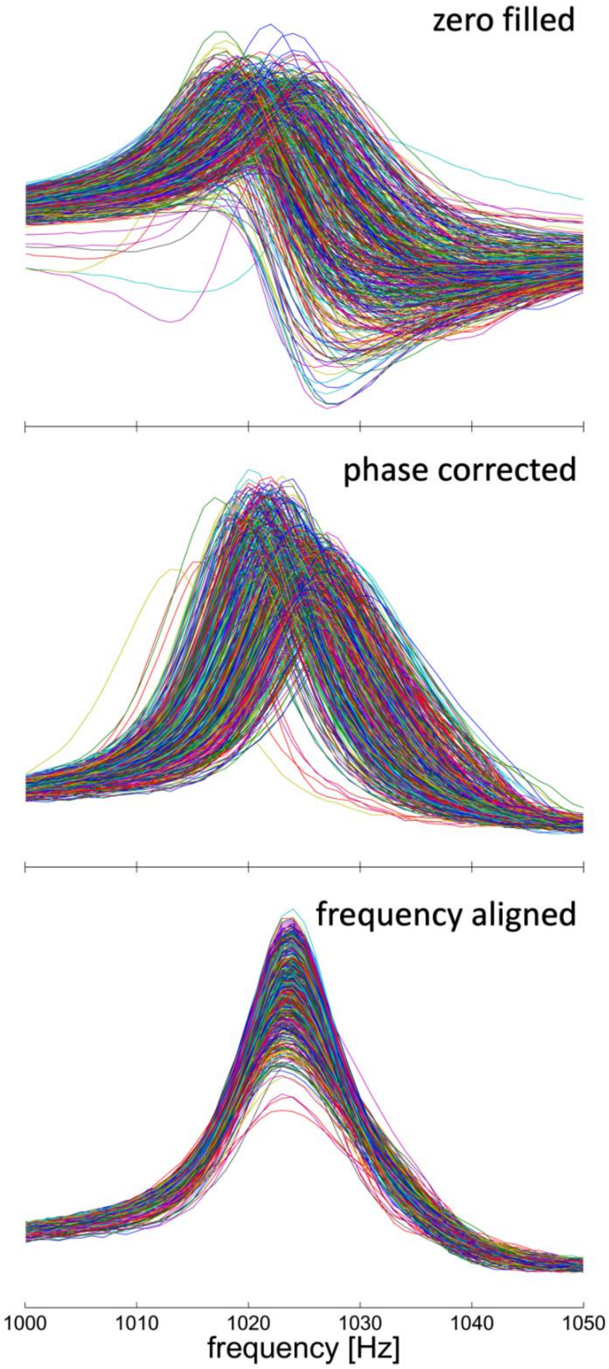

Figure S3.1: Effect of post-processing*: Water peaks of all 512 individual measurements from one volunteer are shown at different stages of post-processing. Top panel: only zero filled; middle panel: zero filled and phase corrected; bottom panel: zero filled, phase corrected and frequency aligned. Zero filling in the time domain leads to an interpolation of data in the frequency domain. It can be seen, that phase correction of each single measurement avoids incoherent averaging due to phase cancellation and frequency alignment prevents artificial peak broadening due to dynamic frequency drifts.*

4. Fitting errors due to incomplete model and internal algorithm constraints?

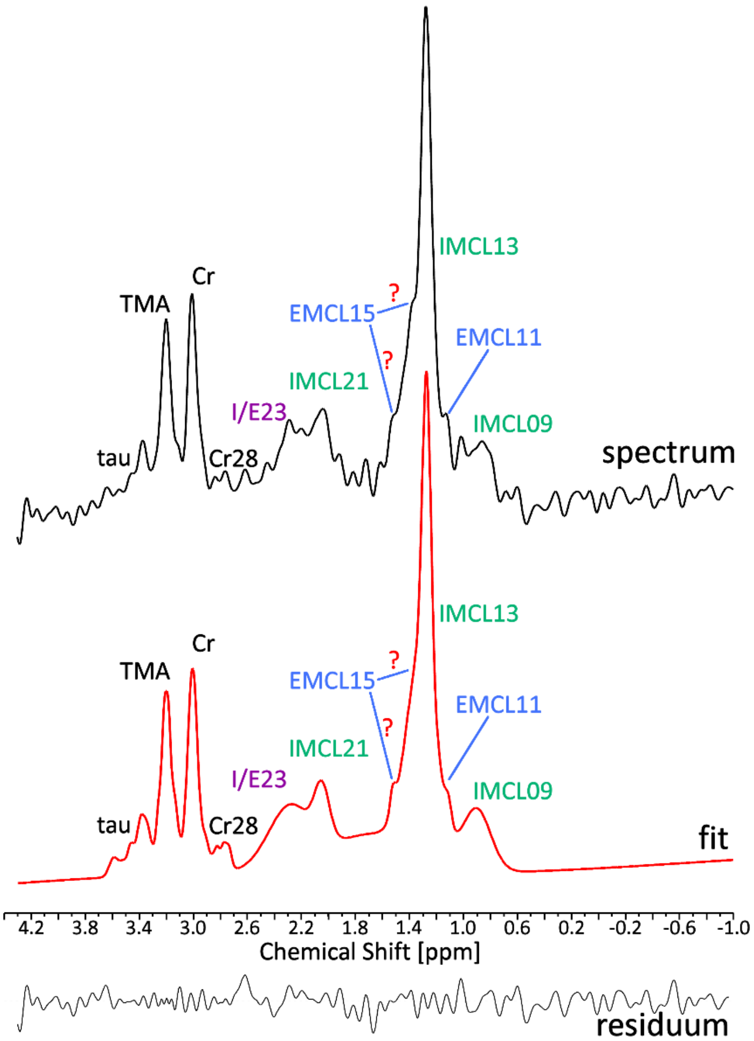

Figure S4.1: Spectrum and fit from the interventricular septum of volunteer 3*.* *Although only 448 averages were acquired in this volunteer, the SNR of this spectrum is sufficiently high for proper analysis. From the theory, the chemical shift difference between EMCL15 and IMCL13 is estimated as* $\Delta\omega_{EMCL} \approx 0.15\, ppm$*, placing the resonance line at approximately 1.39 ppm. A small shoulder can be seen on the IMCL13 peak at that point; however, LCModel fits the EMCL15 resonance line to approximately 1.53 ppm, with an amplitude that is on the order of magnitude of the noise level. The two spectral features in question have been highlighted with red question marks. After closer investigation it became clear that the frequency shift parameters in LCModel are constrained, to avoid peaks being fit too far from their nominal position. While this makes sense for peaks with a fixed resonance frequency, the peak shifts allowed by LCModel clearly restrict the fit of resonance frequencies that shift due to fibre angulation differences with respect to* B_0 *like the EMCL signals. Indeed, if the frequency shift of the EMCL signals relative to IMCL gets too small, the two peaks are not resolvable, independent of SNR levels or linewidths. The two spectra exhibiting these features were, hence, excluded from further analysis involving the chemical shift of EMCL resonances, as well as from lipid quantification.*

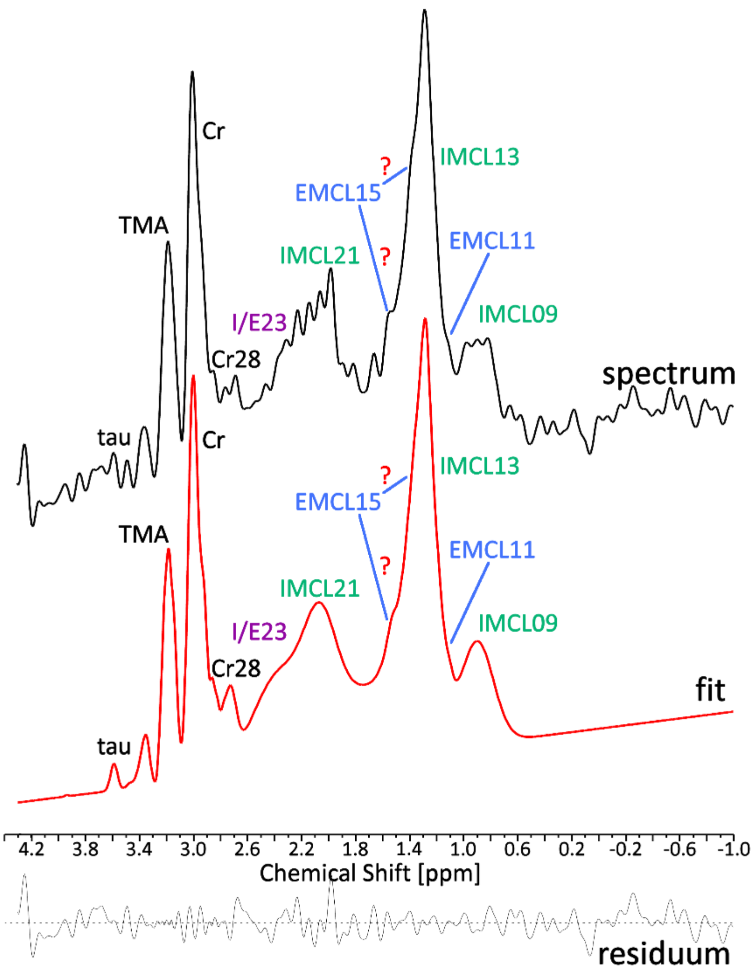

Figure S4.2: Spectrum and fit from the interventricular septum of volunteer 10*. A total of 768 averages were acquired in this volunteer. The two spectral features in question of being the signal from the EMCL15 component have been highlighted with red question marks, with the higher frequency one being that identified by LCModel and the lower frequency one the theoretical position of EMCL15, as calculated from the voxel angle. Since the IMCL13 peak is also shifted slightly from its nominal position at 1.3 ppm, it is possible that the resonance identified by LCModel as EMCL15 actually stems from an IMCL component which resonates at 1.6 ppm, but is not incorporated into the model used to fit the data by LCModel.*

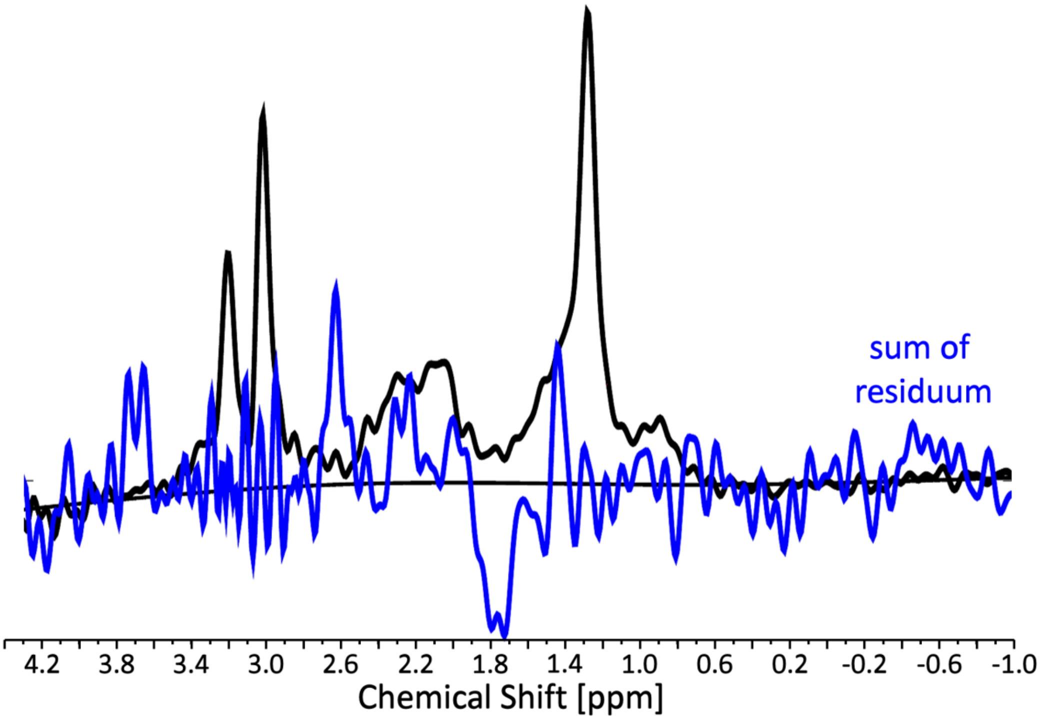

Figure S4.3: Sum of the residuals from the fitted spectra*. The residuals after fitting of the spectrum of each volunteer were summed up (spectra excluded from analysis due to not properly approximated frequency shift of EMCL were not considered). For reference a measured spectrum is also plotted in black. It can be seen that several spectral features are not captured by the underlying fit model, e.g. a peak at approximately 2.7 ppm, which might stem from polyunsaturated fatty acids. Moreover, a significant dip can be seen between 1.6 ppm and 2.0 ppm, which suggests that the employed basis functions for IMCL21 and I/E23 are too broad to be able to capture the more complex spectral structure of the underlying spectral features, which then results in this kind of 'overfitting'.*

5. Quantification

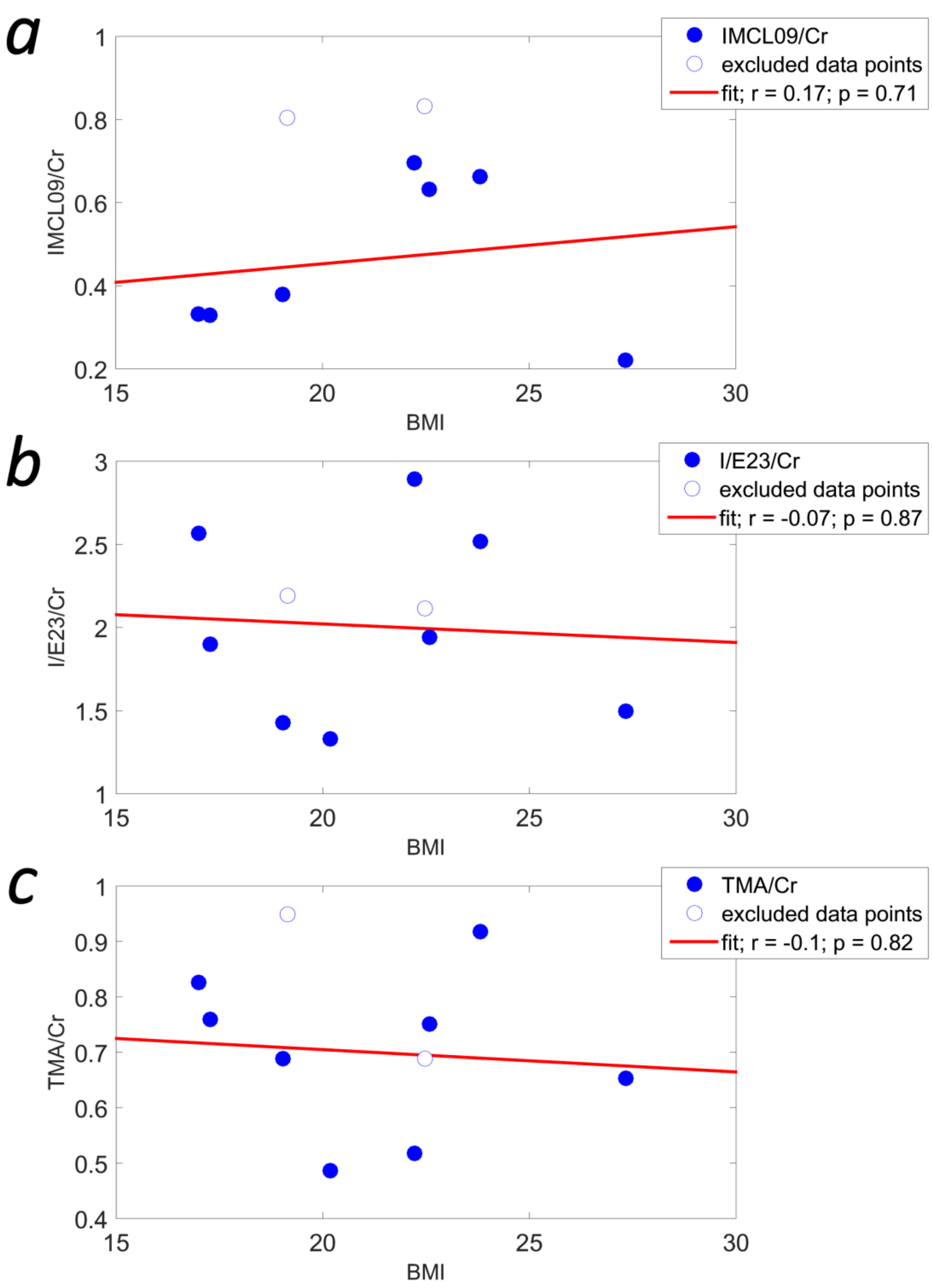

Figure S5.1: Metabolite/Cr ratios plotted versus BMI. IMCL09/Cr (a), I/E23/Cr (b), and TMA/Cr (c) plotted versus the BMI of the volunteers. Data points that were excluded from analysis are shown as open circles. Linear regressions are displayed as red line and Pearson regression coefficients, *r,* and significance values, *p,* are given for each data set. It can be seen that none of the here shown metabolite/Cr ratios correlates with BMI. However, since we know that the fit model used by LCModel is incomplete (namely, an IMCL moiety with its resonance frequency at 2.3 ppm is missing, and therefore IMCL23 and EMCL23 can only be fitted together), it is possible that future studies will uncover significant correlations, especially between the different lipid moieties and BMI.

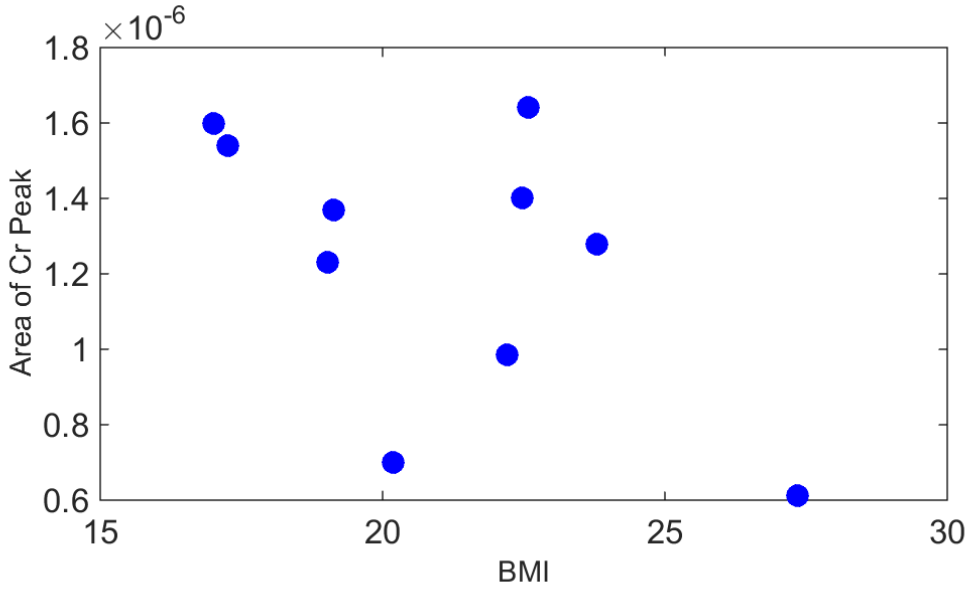

Figure S5.2: Area under the creatine peak plotted versus BMI. While the area under the creatine peak varies between different volunteers, there is no obvious trend or correlation with the volunteers' BMIs. Hence, correlations found between metabolite/Cr ratios are likely to originate from metabolites correlating with the volunteers' BMIs.

References:

1 Boesch, C., Slotboom, J., Hoppeler, H. & Kreis, R. In Vivo Determination of Intra-Myocellular Lipids in Human Muscle by Means of Localized 1H-MR-Spectroscopy. *Magnetic resonance in medicine* **37**, 484-493 (1997).

2 Nielles-Vallespin, S. *et al.* Assessment of Myocardial Microstructure Dynamics by In Vivo Diffusion Tensor Cardiac Magnetic Resonance. *Journal of the American College of Cardiology* **69**, 661-676 (2017).

3 Stoeck, C. T. *et al.* Dual-Phase Cardiac Diffusion Tensor Imaging with Strain Correction. *PLOS One* **9**, e107159 (2014).

4 Stoeck, C. T. *et al.* Direct Comparison of In Vivo Versus Postmortem Second-Order Motion-Compensated Cardiac Diffusion Tensor Imaging. *Magnetic resonance in medicine*, doi:10.1002/mrm.26871 (2017).

5 Edden, R. A., Schar, M., Hillis, A. E. & Barker, P. B. Optimized detection of lactate at high fields using inner volume saturation. *Magnetic resonance in medicine* **56**, 912-917, doi:10.1002/mrm.21030 (2006).
